# Aggregation Mode, Host‐Guest Chemistry in Water, and Extraction Capability of an Uncharged, Water‐Soluble, Liquid Pillar[5]arene Derivative

**DOI:** 10.1002/open.202100206

**Published:** 2021-11-03

**Authors:** Inbar Horin, Ori Shalev, Yoram Cohen

**Affiliations:** ^1^ School of Chemistry, Sackler Faculty of Exact Sciences Tel Aviv University Ramat Aviv 69978 Tel Aviv Israel

**Keywords:** extraction, host-guest complexes, liquid host, pillar[5]arene, water-soluble

## Abstract

An uncharged, water‐soluble per‐ethylene‐glycol pillar[5]arene derivative (**1**) was synthesized and its aggregation mode, host‐guest chemistry in water and extraction ability was explored. Compound **1** is a liquid at room temperature; in water, limited self‐aggregation occurred at high concentrations as deduced from diffusion NMR and dynamic light scattering. Compound **1** forms pseudo‐rotaxane‐like 1 : 1 host‐guest complexes with 1,ω‐di‐substituted alkanes with association constants on the order of 10^3^–10^4^ m ^−1^. Interestingly, NMR experiments showed that the guest location relative to the host ring system differs among the different complexes. In proof‐of‐concept experiments, compound **1** was shown to extract structurally related organic compounds from benzene into water with significant selectivity. Compound **1**, which is a liquid at room temperature and has only limited interactions with its side arms, can, in principle, be regarded as a complement to or as a kind of type I porous liquid.

## Graphical abstract

**A liquid host** based on a per‐ethylene‐glycol pillar[5]arene derivative (**1**) was prepared and its self‐aggregation, host‐guest chemistry in water and extraction capabilities were studied and evaluated. **1** forms pseudo‐rotaxane‐like complexes with 1,ω‐di‐substituted alkanes, showing K_a_ values in the range of 10^3^–10^4^ M^−1^. Host **1** can extract such alkanes with 6–7 fold selectivity and since the interaction with its rim side‐chains is weak, **1** can be regarded as a complement to or as a sort of type 1 porous liquid.

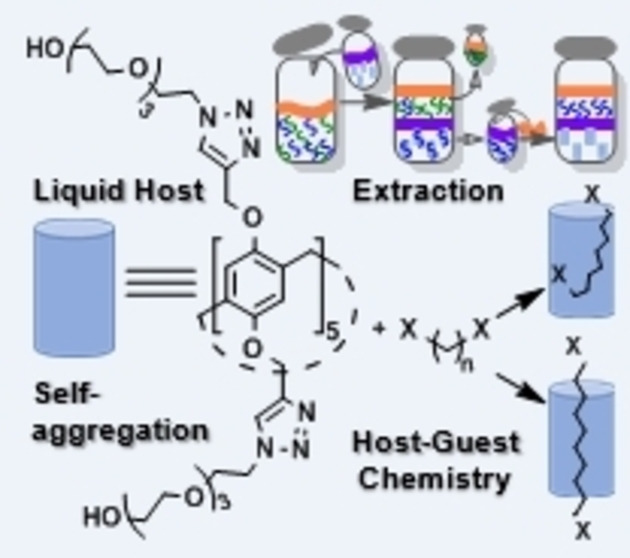

The Nobel Prizes awarded to Pedersen, Cram, and Lehn in 1987 and in 2016 to Stoddart, Feringa, and Sauvage testify for the central role of host‐guest and supramolecular chemistry in the modern chemical sciences.[^1^, ^2^, ^3^, ^4^, ^5^, ^6^] In the last decades, intriguing and fascinating host‐guest and supramolecular systems have been produced and applied in different fields.[^7^, ^8^] In many of these systems, macrocyclic hosts are essential building blocks. Such systems have been used for sensing, in catalysis, and for the preparation of new materials such as supramolecular polymers and gels.[^9^, ^10^, ^11^, ^12^, ^13^, ^14^, ^15^, ^16^, ^17^, ^18^] As Nature operates in water, it is not surprising that host‐guest and supramolecular chemistry in water have become the focus of research.[^19^, ^20^, ^21^, ^22^, ^23^, ^24^] This occurred due to the introduction of new families of macrocycles, some of which are water‐soluble (like cucurbiturils)[^25^, ^26^] and others that can be made water‐soluble relatively easily (like calixarenes and pillararenes).[^27^, ^28^] Some of these water‐soluble host‐guest and supramolecular systems are capable of functioning as drug delivery systems and as diagnostic agents.[^29^, ^30^, ^31^, ^32^]

Pillararenes, first synthesized by Ogoshi in 2008,^[33]^ have cylindrical structures and have become widely used building blocks for supramolecular chemistry in the last decade.[^34^, ^35^, ^36^] Pillararenes have been used for sensing and separation, as platforms for new materials, as drug delivery systems, and more.[^37^, ^38^, ^39^, ^40^, ^41^, ^42^, ^43^, ^44^, ^45^, ^46^, ^47^, ^48^] This occurred, partially, since both organic‐ and more importantly water‐soluble pillararenes, having good host‐guest capabilities, can be constructed relatively easily.[^34^, ^35^, ^36^, ^37^, ^38^, ^39^, ^40^, ^41^, ^42^, ^43^, ^44^, ^45^, ^46^, ^47^, ^48^] Most of the water‐soluble pillararenes prepared to date have been constructed by substituting the pillararene core with charged moieties. Indeed, uncharged water‐soluble pillararene species are scarce, and those that are liquid at room temperature are even more rare.[^49^, ^50^, ^51^] Recently, we demonstrated that pillararene derivatives can be used as inhibitors of biofilm formation,[^52^, ^53^] as sensors for xenon,[^54^, ^55^] and as platforms for the preparation of supramolecular organogels,[^56^, ^57^] and hydrogen bond‐based supramolecular boxes in water.[^58^, ^59^] Herein, we synthesized and studied the aggregation mode of a per‐ethylene‐glycol pillar[5]arene derivative, namely compound **1** (Figure 1), in different solvents and its host‐guest chemistry in water. We also evaluated its ability to extract organic compounds from benzene into water and its interaction with tetra‐ethylene glycol, that is, its side arms. Interestingly, host **1** is a liquid at 25 °C, that is, room temperature.

**Figure 1:**
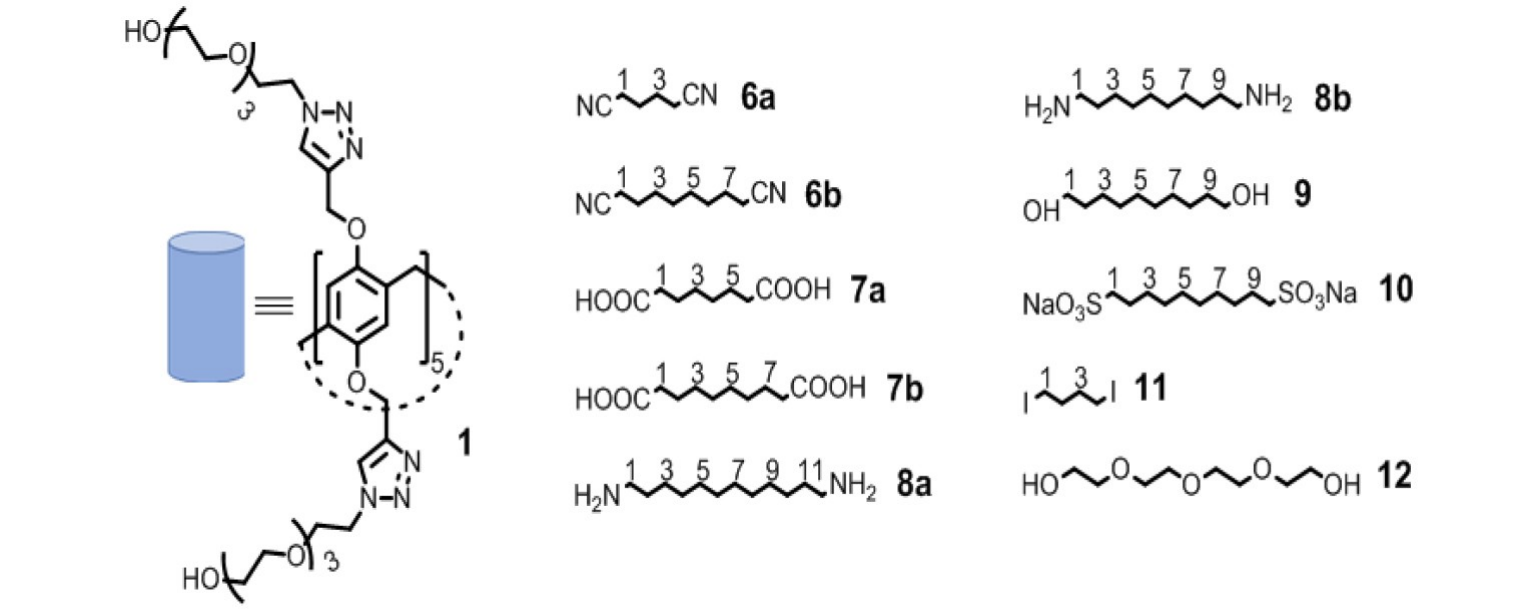
Molecular structures of host **1** and guests **6**–**12**.

The synthesis of **1** (see Scheme S1, Supporting Information) started with the alkylation of hydroquinone with propargyl bromide to give compound **2**. Then, compound **2** was treated with formaldehyde and BF_3_‐etherate to give pillar[5]arene **3**. Finally, **3** was reacted, in a Cu‐catalyzed click reaction, with tetra‐ethylene glycol mono‐azide **4**, prepared from tetra‐ethylene glycol **12**, to yield compound **1**.

The full characterizations of compounds **1**–**4** are given in Figures S1–S12 (Supporting Information). Compound **1** is highly soluble in water and in a wide range of organic solvents (Figure 2 and Figures S7–S12). The ^1^H NMR spectra of **1** are different in the various solvents tested, with broader lines in CDCl_3_ and D_2_O compared to DMSO‐d_6_, acetone‐d_6_, and CD_3_OD. This seems to suggest that compound **1** self‐aggregates, to some extent, in chloroform and water.

**Figure 2:**
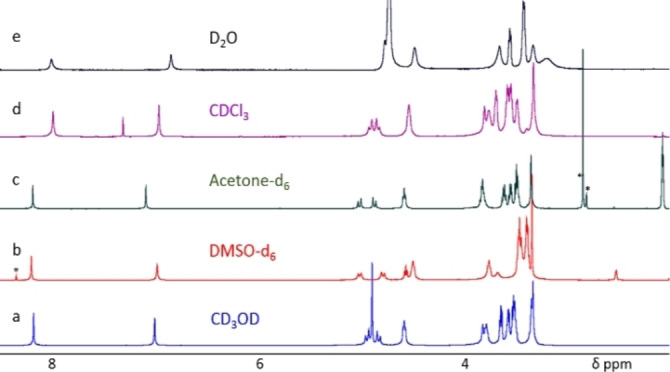
^1^H NMR spectra (400 MHz, 298 K) of compound **1** (3 mm) in a) CD_3_OD, b) DMSO‐d_6_, c) acetone‐d_6_, d) CDCl_3_, and e) D_2_O (* residual water or chloroform).

To explore the aggregation properties of compound **1**, we turned to diffusion NMR spectroscopy[^60^, ^61^] and the data is presented in Table S1 in the Supporting Information. From this data, the Stokes‐Einstein hydrodynamic radii (r_s_) of **1** in CDCl_3_ (3 mm), D_2_O (3 mm, 0.4 mm) and acetone‐d_6_ (3 mm) were compiled to be 1.22, 1.63, 1.53 and 1.21 nm, respectively (see section 8 of the Supporting Information). The hydrodynamic radii obtained from dynamic light scattering (DLS) in D_2_O (Figure S40) were 1.7 and 1.4 nm at 3 mm and 0.4 mm concentrations of **1**, respectively, in agreement with the values extracted from diffusion NMR spectroscopy. Based on these results, we concluded that some self‐aggregation of **1** does occur in water. To further evaluate self‐aggregation, we measured the diffusion coefficients of **1** in water at even higher concentrations (Table S1). Although some self‐aggregation of **1** occurred in water, the extent was low even at a high concentration of 50 mm.

Next, we studied whether compound **1** is able to host different 1,ω‐di‐functionalized alkanes such as compounds **6**–**10** (Figure 1) in water. The ^1^H NMR spectra of 1 : 2 aqueous solutions of **1** with compounds **6 a**, **6 b**, **7 a**, **7 b**, **8 a**, and **10** are presented in Figure 3. These data demonstrate that such complexes are formed and that there is a slow exchange between the complexed and un‐complexed guests on the ^1^H NMR chemical shift timescale.

**Figure 3:**
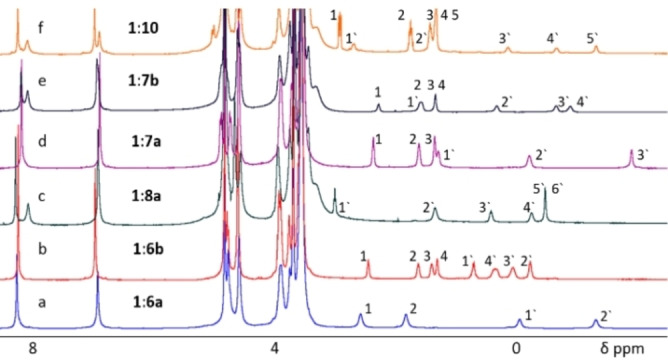
^1^H NMR spectra (D_2_O, 298 K, 400 or 500 MHz) of 10 mm of **1** and 20 mm of guests: a) **6 a**, b) **6 b**, c) **8 a**, d) **7 a**, e) **7 b**, and f) **10**. Note, however, that the effective concentrations of **7 a**, **7 b**, and **8 a** were only 19, 7, and 5 mm, respectively, due to solubility issues. The numbered positions of guests are depicted in Figure 1. Numbers without prime symbols mark peaks of unbound guests.

One‐ and two‐dimensional ^1^H NMR spectra of the complexes of **1** with the different guests, that is, **6**–**10**, were collected for an unequivocal assignment of the different protons of the free and complexed guests (Figure 3 and Figures S13–S37). The formation of the complexes of **1** with the different guests was deduced from the up‐field shifted signals in the ^1^H NMR spectra of these solutions. For some guests, complex formation was corroborated by diffusion NMR spectroscopy as previously described.[^62^, ^63^, ^64^, ^65^] Diffusion data showed that the host and guest in these complexes diffuse as a single entity (Table 1), as expected.[^62^, ^63^, ^64^, ^65^] Heating was required to obtain the complexes of **1** with compounds **7 a**, **8 a**, **9**, and **10**. All other complexes were formed rapidly at room temperature.

**Table 1:** Diffusion coefficient (D) at 298 K of the different species in 1 : 2 D_2_O solutions of host **1** with **6 a**, **6 b**, **7 a**, and **8 a** as guests

Sample Content			
	Compound **1**	Encapsulated Guest	Free Guest
**1**:**6 a** (10 : 20 mm)	0.12±0.01	0.13±0.01	0.41±0.02
**1**:**6 b** (10 : 20 mm)	0.12±0.01	0.12±0.01	0.50±0.01
**1**:**7 a** (10 : 20 mm)	0.12±0.01	0.17±0.01	0.30±0.01
**1**:**8 a** (10 : 20 mm)	0.13±0.01	0.14±0.01	NA

[a] Diffusion coefficients are given as mean±standard error of the mean of at least three independent measurements.

Different guests may adopt different spatial arrangements and may interact with different sites in the host cavity upon complexation. The different possible spatial organizations of 1,ω‐bi‐functionalized alkanes in a tubular system like a pillar[5]arene are presented in Figure 4. The most plausible possibilities within the pillar[5]arene cavity are the pseudo‐rotaxane‐like structures shown in Figure 4a and Figure 4b; structures like those shown in Figures 4c and 4d are more likely to occur in concave systems.[^66^, ^67^, ^68^, ^69^]

**Figure 4:**
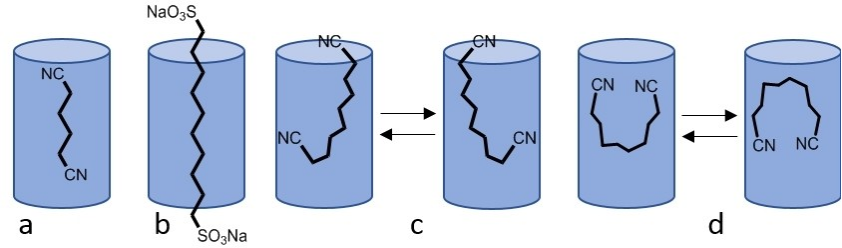
A cartoon representation of some of the plausible spatial arrangements of 1,ω‐bi‐functionalized alkanes in a pillar‐shaped host such as **1**.

In complexes with compound **1**, the alkyl chain of 1,ω‐di‐substituted alkanes may, in principle, adopt an extended, bended, or even coiled form. For example, we found that the short adiponitrile guest (**6 a**) forms a pseudo‐rotaxane‐like type complex with **1** as shown in Figure 4a. This was deduced from the ^1^H NMR data presented in Figure 3a and Figures S13–S16 and from the finding that all the protons of guest **6 a** show significant high‐field shifts. These shifts are on the order of 3.2 ppm for protons at positions 2 and 3 and of 2.6 ppm for protons at positions 1 and 4. This implies that the protons at positions 2 and 3 occupy the space above the aromatic rings of the pillararene core, experiencing nearly the maximal ring current effect of the aromatic core of **1**. The significant 2.6 ppm high‐field shift of protons at positions 1 and 4 implies that these protons are also significantly impacted by the ring current effects.

The much longer guest **10**, which has two very polar end groups, adopts the pseudo‐rotaxane structure shown in Figure 4b. Upon complexation, the protons of compound **10** at positions 5 and 6 show high‐field shifts of 2.7 ppm, and those at positions 4 and 7 show high‐field shifts of 2.0 ppm, whereas those at positions 2 and 9 are shifted by only 0.4 ppm and those at positions 1 and 10 are shifted by only 0.2 ppm. For compound **10**, the difference between the largest and smallest change in chemical shift upon complexation (ΔΔδ_H_) is 2.5 ppm. All these observations are in line with the formation of a pseudo‐rotaxane type complex like the one presented in Figure 4b, where the long alkyl chain extends to both sides of the pillararene core.

Based on the assigned ^1^H NMR spectra (Figure 3), we concluded that guests **7 a**, **7 b**, and **8 a** also form complexes of the type presented in Figure 4b. The one guest that appears to behave differently is sebaconitrile (**6 b**). For **6 b**, the largest and the smallest up‐field shifts upon complexation are 1.9 ppm (for protons at positions 2 and 7) and 1.0 ppm (for protons at positions 4 and 5), implying that ΔΔδ_H_ is only 0.9 ppm. These results, together with the fact that the changes in the chemical shifts upon complexation (Δδ_H_) are small and that the extents of the shifts do not correlate with the position along the chain – as they do for other guests – imply that compound **6 b** adopts the structure depicted in Figure 4c when complexed by compound **1**.

In addition, the 2D NMR data presented in Figure 5 and Figures S19–S20 and S36–S37 strengthens our hypotheses that in complex with compound **1**, compound **10** adopts the extended form, whereas compound **6 b** is kinked. NMR spectroscopy enabled us to estimate the association constants (K_a_) of the complexes of **1** with **6 a** and **6 b**. The K_a_ values were found to be in the range of 10^3^–10^4^ m ^−1^ (see section 9 of the Supporting Information). These are high affinity complexes for uncharged host‐guest complexes in water and are similar to the K_a_ value for the methylated analogue of **1** reported by Wu et al..^[51]^ However, in this case, fast exchange on the ^1^H NMR chemical shift timescale was observed between the bound and the free guest molecules.

**Figure 5:**
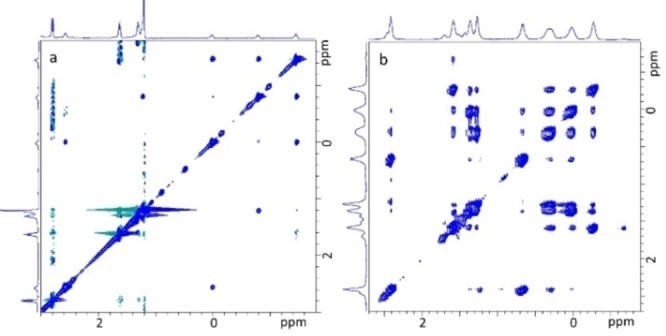
Excerpt of the ^1^H‐NOESY spectrum (500 MHz, D_2_O, 298 K) of complexes of 10 mm of pillar[5]arene **1** with a) 10 mm of **10** and b) 20 mm of **6 b**.

As compound **1** is uncharged and soluble in organic solvents as well as in water, it has potential as a reagent for extraction, transport, and separation. Since **1** has extremely low solubility in benzene, we used a benzene−water system to evaluate the ability of compound **1** to extract organic compounds from the organic phase into water (Figure 6). For these proof‐of‐concept experiments, we selected three structurally related compounds, adiponitrile (**6 a**), 1,4‐diiodo‐butane (**11**), and sebaconitrile (**6 b**) (Figure 1). The results are summarized in Table S3 and Figures S41–S52. We investigated the extraction ability of **1** from 1 : 1 solutions of **6 a** with **6 b** and with **11**, which enabled us to examine the extraction selectivity of host **1** for guests that differ in chain length (**6 a** versus **6 b**) and in their functional end‐groups (**6 a** versus **11**). The results indicate that there is 6–7‐fold selectivity in the extraction of guest **6 a** compared to **6 b** and **11**. In addition, **1** bound to **6 b** or to **11** could be regenerated by a simple washing procedure; this was not the case for **6 a** (see Table S3).

**Figure 6:**
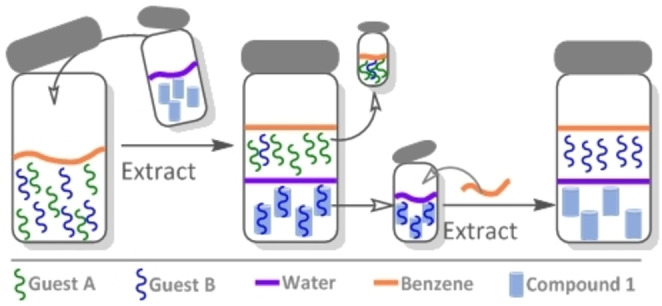
Cartoon representation of the extraction cycle performed with compound **1** and different guest molecules.

Compound **1** is a liquid at room temperature and as such can, in principle, be regarded as a complement to or as a type I porous liquid.^[70]^ Porous liquids have attracted considerable attention in recent years due to their potential application in the field of separation.[^71^, ^72^, ^73^, ^74^, ^75^] To determine whether compound **1** may acts as a porous liquid, we evaluated the interactions of compound **1** with tetra‐ethylene glycol (**12**), its rim side‐arms, using diffusion NMR spectroscopy and DOSY experiments. Because of the very large difference between the molecular weights and the cross sections of these two molecules, the diffusion coefficient should, in principle, be a suitable parameter for monitoring this interaction.[^63^, ^64^, ^65^, ^76^] We measured the diffusion coefficient of **12** in both absence and presence of **1**. The diffusion coefficient of **12** was nearly 3‐fold higher than that of **1** (Figure 7 and Table S2). In the presence of **1**, the diffusion coefficient of **12** was only slightly lower than that of **12** in the absence of **1**. These results imply that the interaction of **12** with **1** is relatively weak, suggesting that the interactions of the side‐arm groups of **1** with its cavity are also relatively weak, leaving room for other potential guests. Thus, compound **1** may act as a complement to or as a sort of a type I porous liquid.

**Figure 7:**
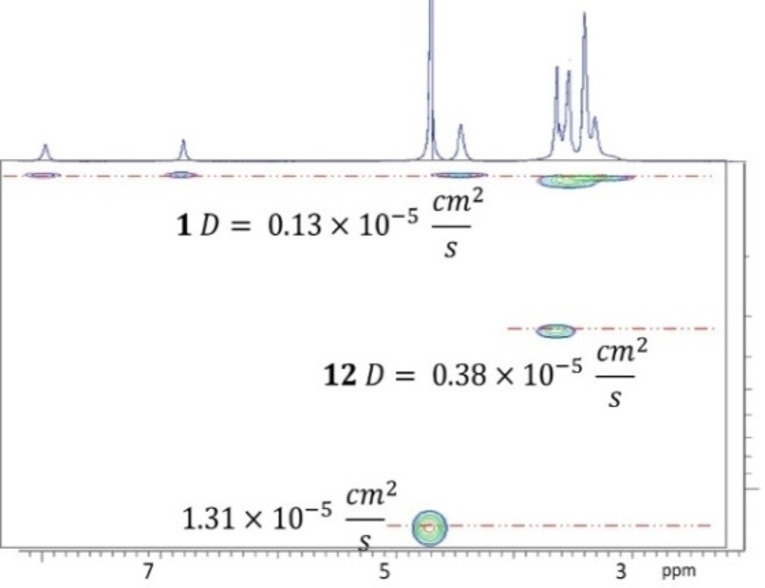
^1^H‐DOSY (500 MHz, 298 K, D_2_O) of a 1 : 1 10 mm solution of **1** and **12**.

To conclude, we synthesized an uncharged, water‐soluble per‐ethylene‐glycol pillar[5]arene derivative **1** and studied its aggregation mode in different solvents and its host‐guest chemistry in water with a series of 1,ω‐bi‐functionalized alkanes. We found, using NMR spectroscopy, that with the exception of **6 b**, the ligands studied form complexes with **1** that have pseudo‐rotaxane‐type structures. The K_a_ values of the host‐guest complexes of **1** with **6 a** and **6 b** were in the order of 10^3^–10^4^ m ^−1^. In proof‐of‐concept experiments, compound **1** efficiently extracted structurally related di‐functionalized alkane guests with 6–7‐fold selectivity. Host **1** is a liquid at room temperature and since the interaction of **1** with tetra‐ethylene glycol, its rim side chains, is relatively weak, compound **1** may operate as a complement to or as a sort of a type I porous liquid.

## Conflict of interest

The authors declare no conflict of interest.

## Supporting information

As a service to our authors and readers, this journal provides supporting information supplied by the authors. Such materials are peer reviewed and may be re‐organized for online delivery, but are not copy‐edited or typeset. Technical support issues arising from supporting information (other than missing files) should be addressed to the authors.

Supporting Information
