# Supplementary material for: Aggregation Mode, Host‐Guest Chemistry in Water, and Extraction Capability of an Uncharged, Water‐Soluble, Liquid Pillar[5]arene Derivative
Source: ChemistryOpen. 2021 Nov 3;10(11):1111–5. doi: 10.1002/open.202100206 (PMC8564886; doi:10.1002/open.202100206)
Supplement: Supplementary file 1 — Supporting Information [file OPEN-10-1111-s001.pdf]

ChemistryOpen

Supporting Information

Aggregation Mode, Host-Guest Chemistry in Water, and Extraction Capability of an Uncharged, Water-Soluble, Liquid Pillar[5]arene Derivative

Inbar Horin, Ori Shalev, and Yoram Cohen*

Supporting Information:

Table of Contents

1. Materials and Methods S2
2. Scheme for the preparation of compound **1** S4
3. Synthesis of compound **1** S4
4. NMR characterization of compounds **1-4** S5
5. ^{1}H-NMR and **1** with different guest molecules S11
6. ^{1}H-NMR of **1** and **12** S24
7. Diffusion NMR and DLS results S25
6. Size extraction from diffusion NMR S27
8. Association constants (K_as) of **1** with different guest complexes S27
9. Extraction experiments S27
10. References S34

1. Materials and Methods

General. Starting materials were purchased from Sigma-Aldrich, Alfa Aesar, TCI, Cambridge Isotope Laboratories, and Bio-Lab Ltd and used as received. Chemical reactions were monitored by TLC (Merck, silica gel 60 F254) and the compounds were purified by SiO_2 flash chromatography (Merck Kieselgel 60). ^{1}H- and ^{13}C-NMR spectra were recorded on 400 and 500 MHz Bruker Avance NMR spectrometers. Chemical shifts (δ) are given in part per millions (ppm), and spin-spin coupling (*J*) in Hz. The chemical shifts are quoted relative to residual HDO signal (at δ 4.79 ppm for the ^{1}H NMR), when the solvent is D_2O, to residual $CHCl_3$ signal (at δ 7.26 ppm for the ^{1}H NMR and 77.2 ppm for the ^{13}C NMR) when the solvent is $CDCl_3$. Abbreviations for multiplicities used: s = singlet, d = doublet, dd = double doublet , t = triplet, q = quartet, quint = quintet, sex = sextet, m = multiplet, br = broad signal. High-resolution electrospray ionization (ESI) mass-spectra were recorded on a Waters Synapt instrument. DLS: All measurements were recorded on a malvern Zetasizer Nano ZS at 298 K.

Diffusion Studies. ^{1}H-diffusion NMR measurements were performed on 11.7T and 9.4T Avance III Bruker NMR spectrometers, operating at 500.16 and 400.13 MHz for ^{1}H, respectively, equipped with a z-gradient system capable of producing maximal gradient pulses of about 50 G/cm. ^{1}H-diffusion NMR experiments were performed using the eddy currents delays (LED) pulse sequence[1]. Sine-shaped pulse gradients, of 4 ms duration, were incremented from 0.7 to 32.2 G/cm (effective gradient strength: G(2/π)) in 10 steps, and the pulse gradient separation was 50 ms. The diffusion coefficients were extracted from: $\ln I/I_0=-\gamma^2\delta^2G^2(2/\pi)^2(\Delta-\delta/4)D=-bD$, where I and I_0 are the echo intensity in the presence and absence of the gradient pulses, respectively, γ is the gyromagnetic ratio, G is the pulse gradient strength, 2/π is a geometrical correction factor due to the sine shape of the pulse gradients used, δ is the duration of the pulse gradient, Δ is the time interval between the leading edges of the pulse gradient used, and D is the diffusion coefficient. The diffusion coefficients were extracted from the slope of the plot of $\ln(I/I_0)$ against the b-values. All diffusion NMR experiments were performed at 298K in triplicates. The given values represent means ± the standard error of the means (SEM). The obtained diffusion coefficients were calibrated using the literature value for HOD diffusion.

The 2D DOSY experiments were performed in the same manner and under the same conditions but with at least 32 gradient values.

2. Scheme for the preparation of compound 1

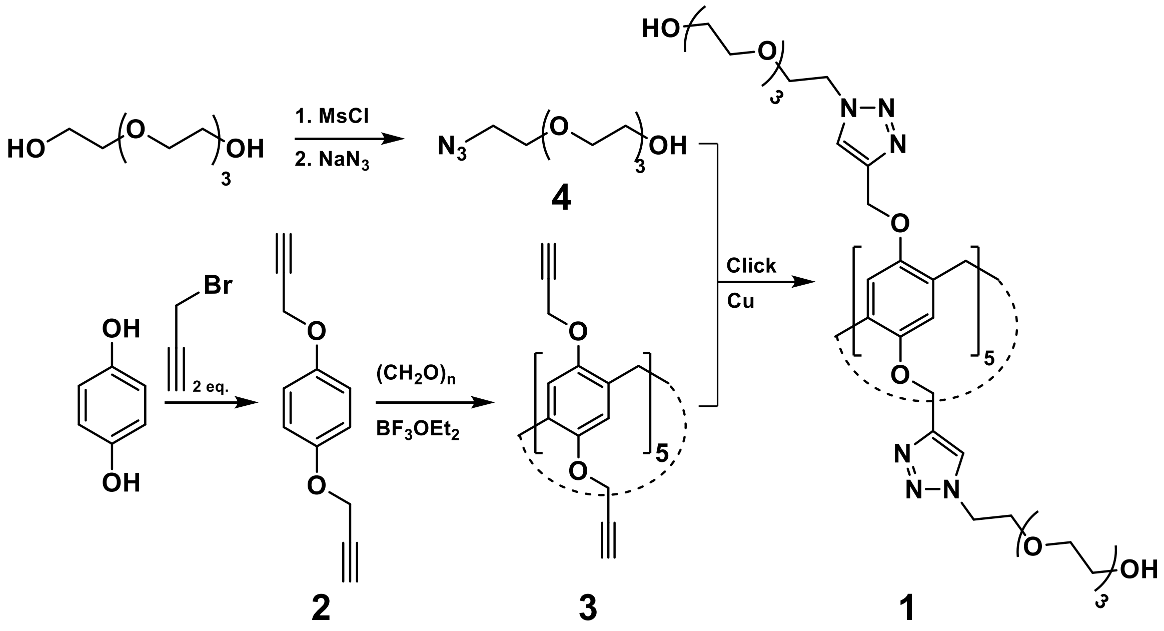

Scheme S1: Full synthetic path for compound **1**.

3. Synthesis of compound 1

Compounds **2**, **3** and **4** - were synthesized and characterized according to the reported procedures.[2,3]

Compound **1:** Compound **4** (1.05 g, 4.5 mmol) was added to a solution of compound **3** (0.4 g, 0.4 mmol), copper sulfate pentahydrate (0.028 g, 0.11 mmol) and ascorbic acid (0.146 g, 0.8 mmol) in DMF (19.8 mL). The mixture was heated to 90 ^{0}C for 24h. The resulting mixture was cooled to RT and evaporated. The resulting solids were dissolved in DCM and washed, twice, with saturated sodium bicarbonate aqueous solution and twice with brine. The resulting product was purified by column chromatography ($CHCl_3$/MeOH) to afford **1** as viscous light brown liquid (0.497 g, 39%). ^{1}H-NMR (400 MHz, Acetone-d-$_6$): δ 8.15 (s, Ar*H*, 10H), 7.05 (s, Ar*H*, 10H), 5.00-4.82 (d, ArOCH_2CNC J = 11 Hz, 20H), 4.55 (t, NCH_2CH_2 J = 5 Hz, 10H), 3.83-3.76 (m, OCH_2CH_2N & ArCH_2Ar, 30H), 3.59-3.45 (m, OCH_2CH_2OCH_2CH_2O, 80H), 3.3 (m, OCH_2CH_2OH, 40H) ppm. ^{13}C NMR (100 MHz, Acetone-d-$_6$): δ 150.3, 144.7, 129.4, 125.2, 115.7, 73.5, 71.0, 70.7, 70.6, 69.9 62.5, 61.9, 54.9, 50.7, 29.5 ppm. HRMS: m/z calcd. for $C_{145}H_{220}N_{30}O_{50}$ $[M+Na]^+$ 3204.5492, found 3204.5508.

4. NMR characterization of compounds 1-4

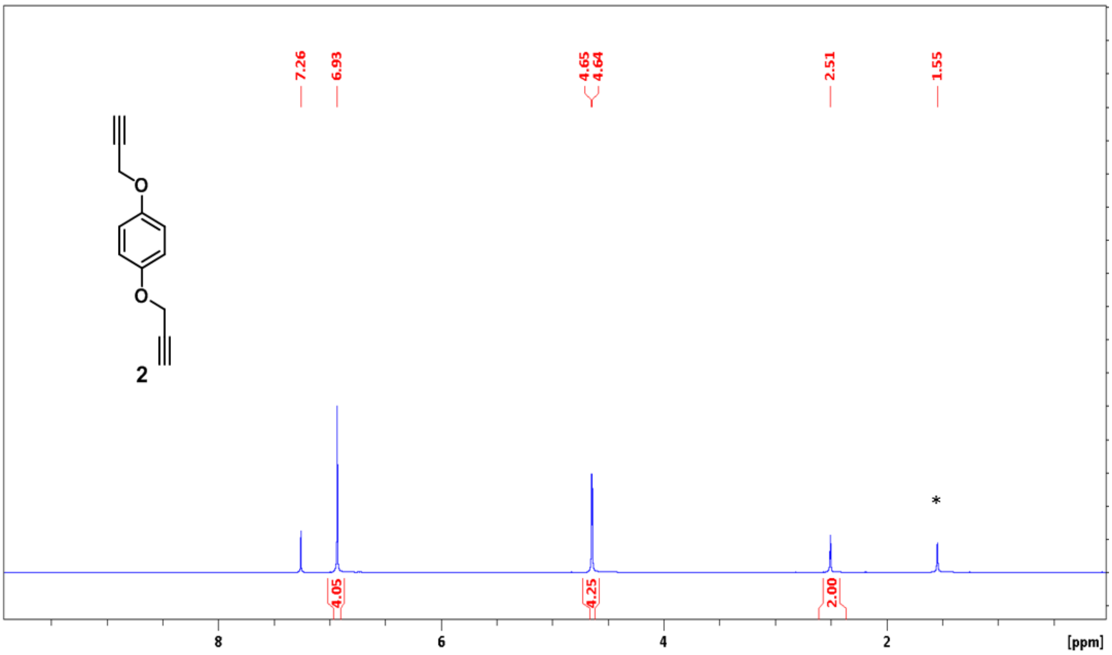

Figure S1. ^{1}H-NMR spectra (400 MHz, 298K) of **2** in $CDCl_3$. * H_2O.

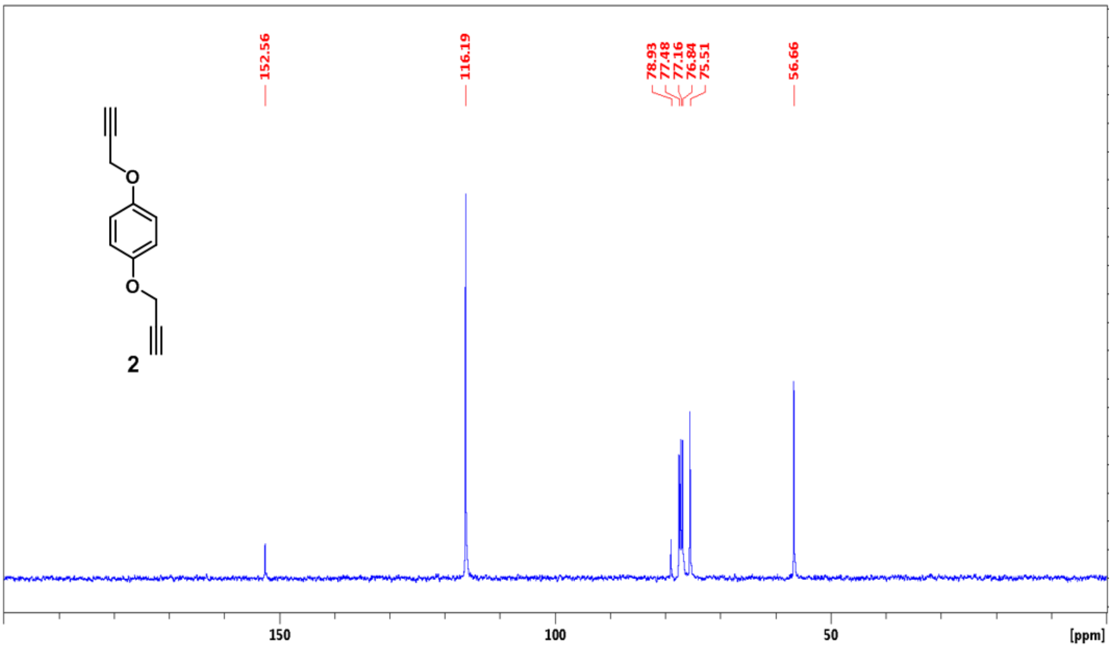

Figure S2. ^{13}C-NMR spectra (100 MHz, 298K) of **2** in $CDCl_3$.

*

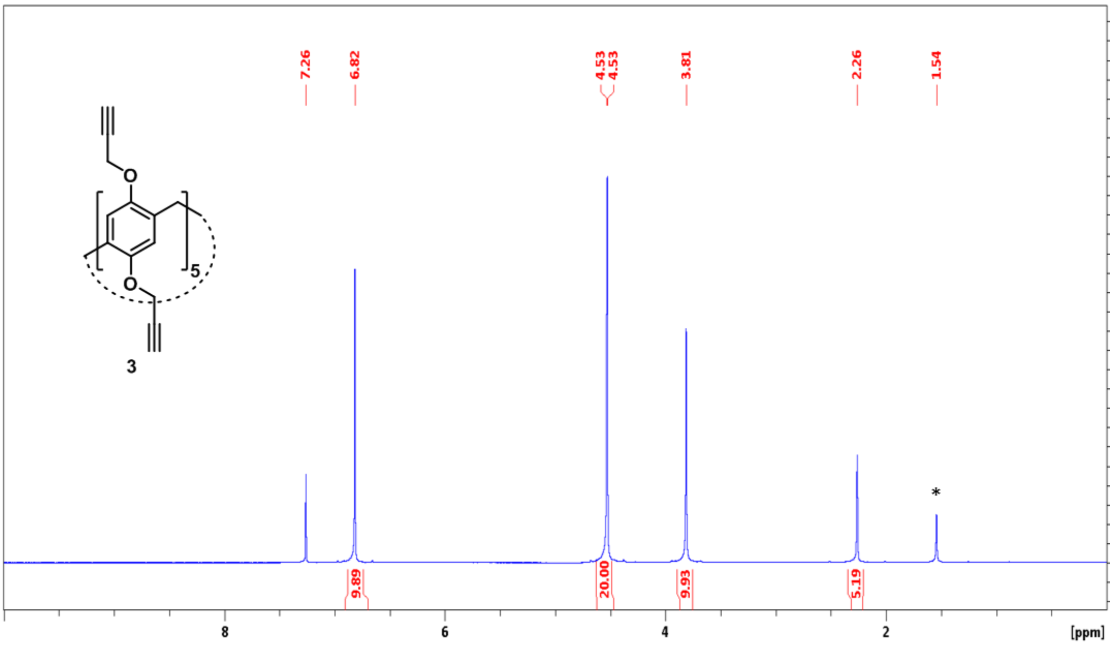

Figure S3. ^{1}H-NMR spectra (500 MHz, 298K) of **3** in $CDCl_3$. * H_2O.

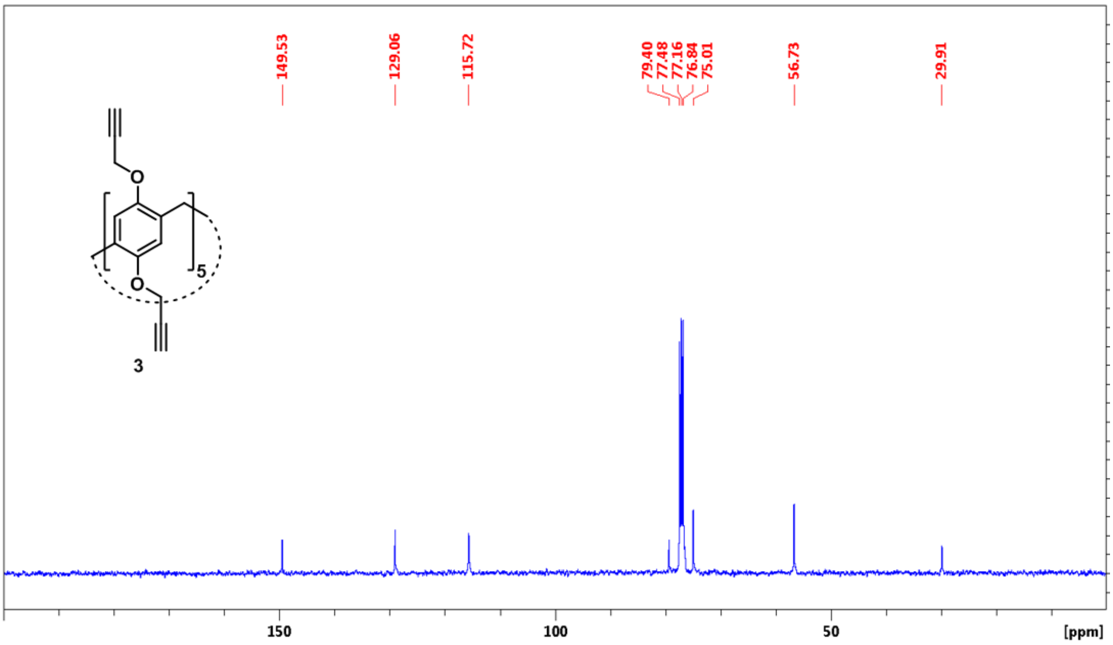

Figure S4. ^{13}C-NMR spectra (100 MHz, 298K) of **3** in $CDCl_3$.

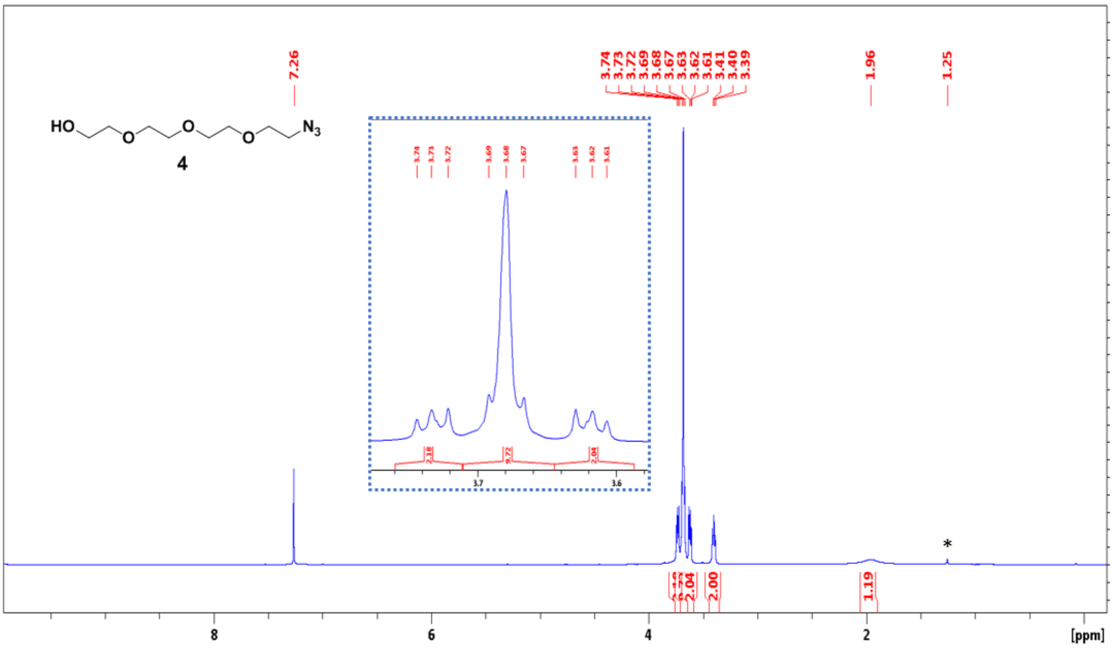

Figure S5. ^{1}H-NMR spectra (400 MHz, 298K) of **4** in $CDCl_3$. * H_2O.

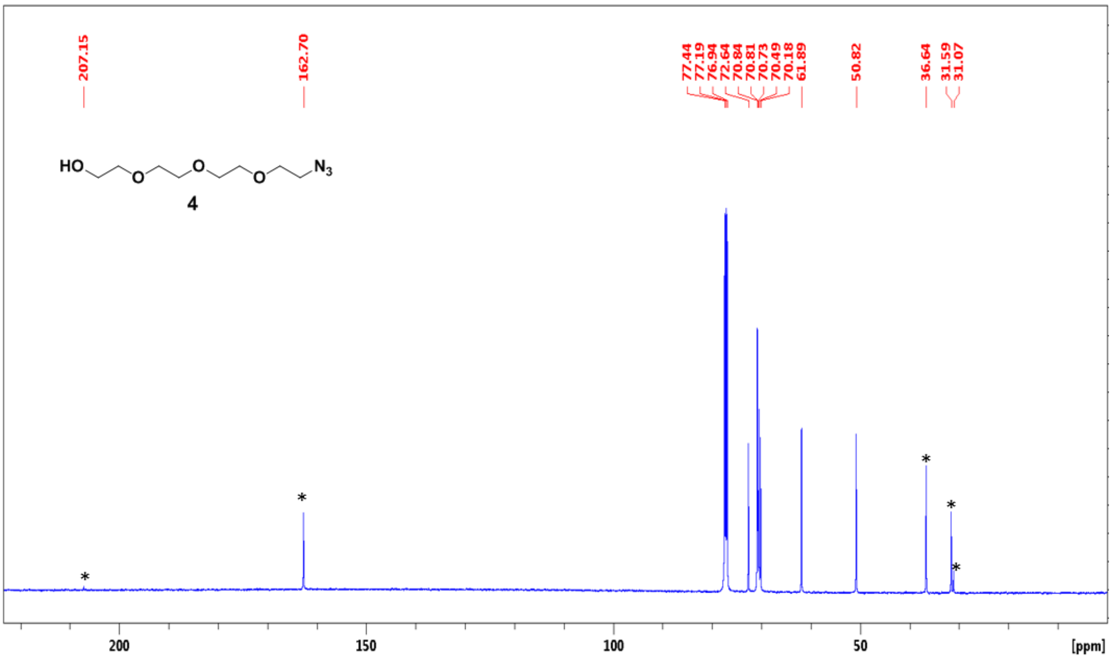

Figure S6. ^{13}C-NMR spectra (125 MHz, 298K) of **4** in $CDCl_3$. * DMF and acetone residues.

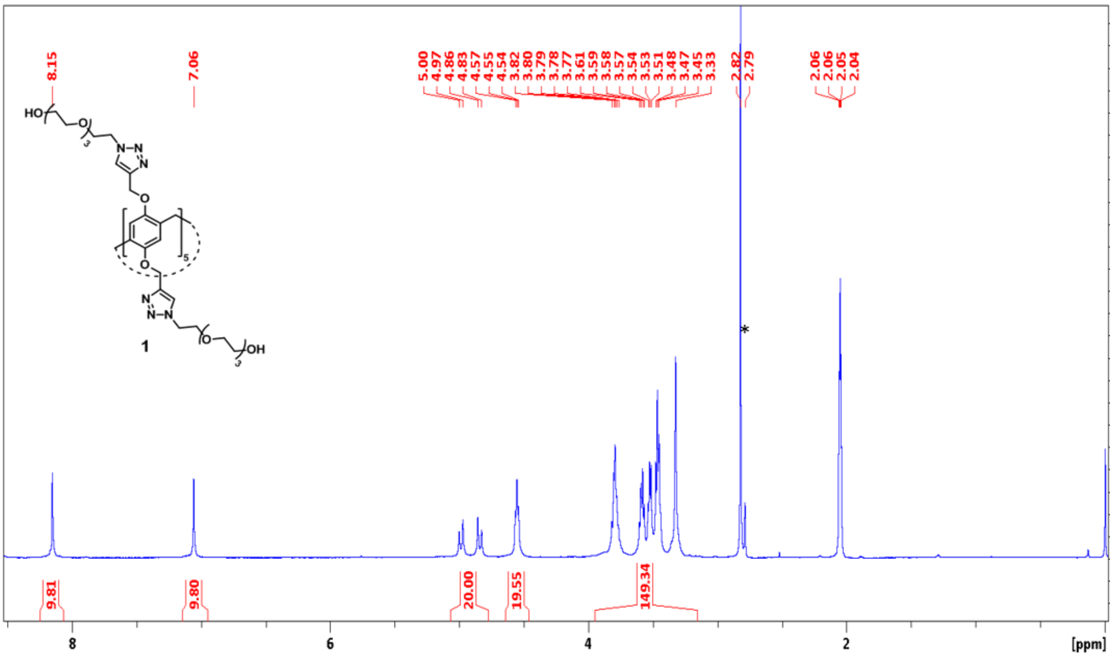

Figure S7. ^{1}H-NMR spectra (400 MHz, 298K) of **1** in Acetone-d_6. * D_2O.

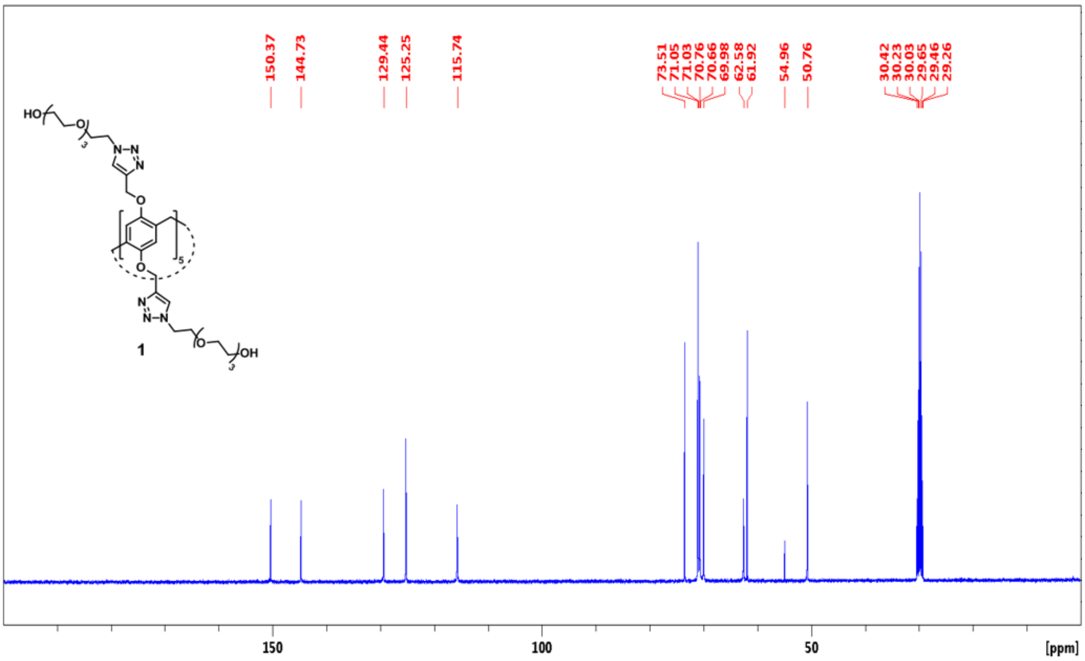

Figure S8. ^{13}C-NMR spectra (100 MHz, 298K) of **1** in Acetone-d_6.

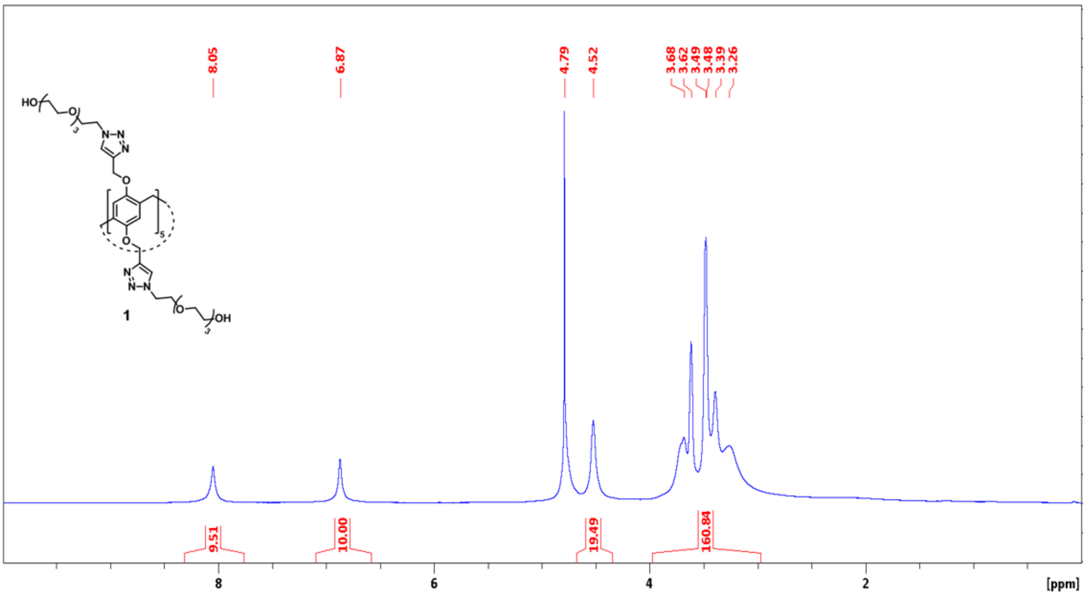

Figure S9. ^{1}H-NMR spectra (400 MHz, 298K) of **1** in D_2O.

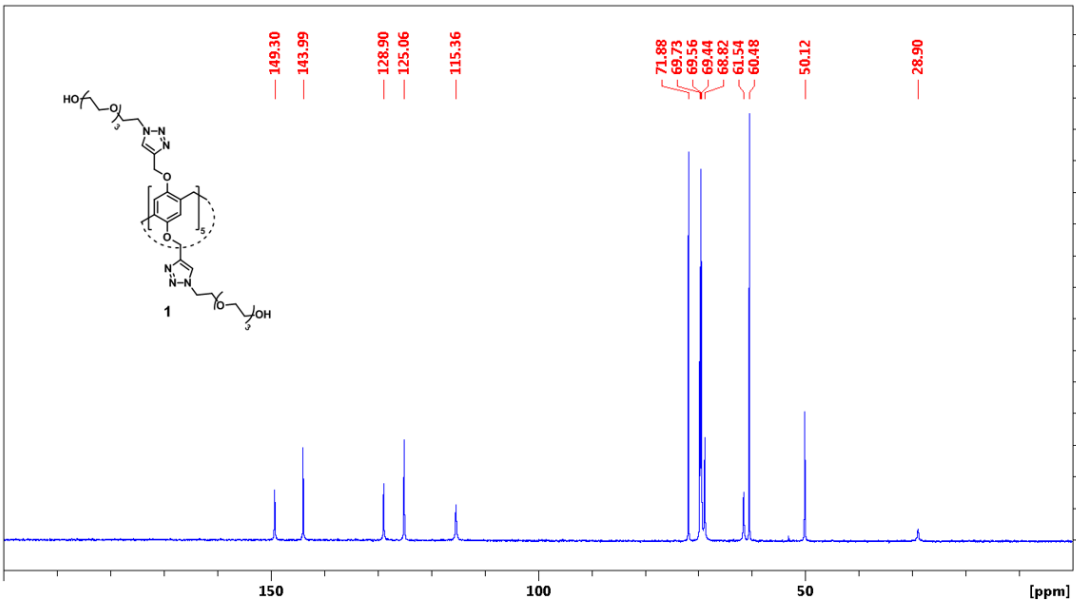

Figure S10. ^{13}C-NMR spectra (125 MHz, 298K) of **1** in D_2O.

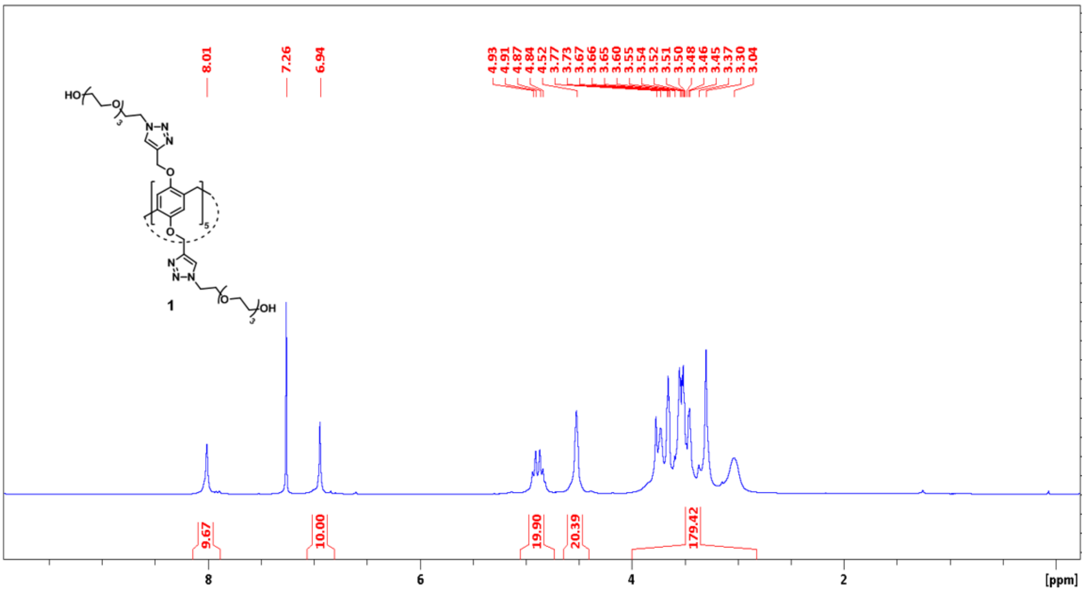

Figure S11. ^{1}H-NMR spectra (400 MHz, 298K) of **1** in $CDCl_3$.

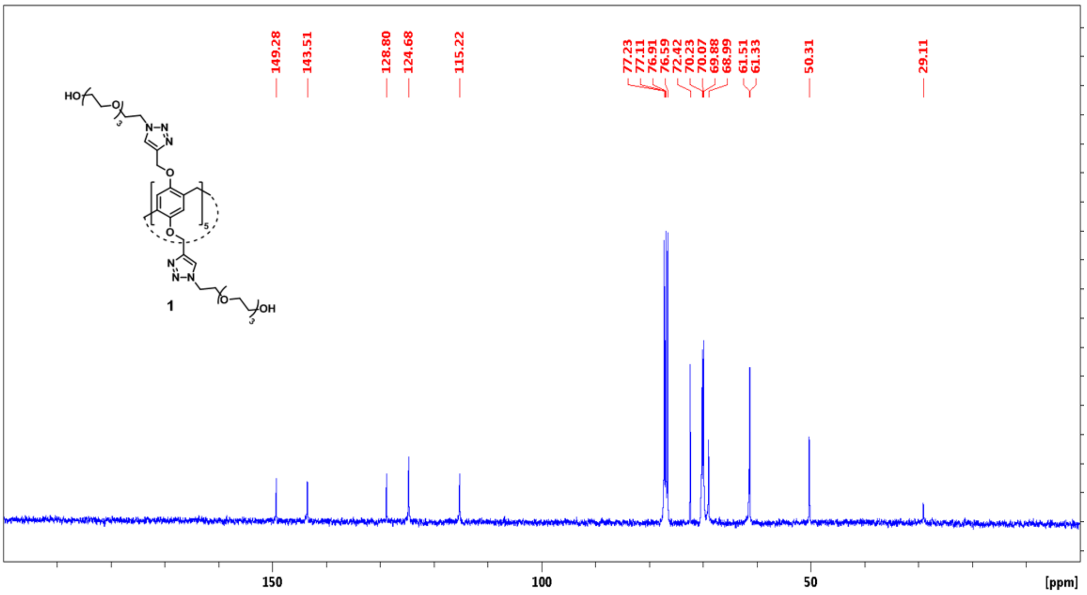

Figure S12. ^{13}C-NMR spectra (100 MHz, 298K) of **1** in $CDCl_3$.

5. ^{1}H-NMR of 1 with different guest molecules

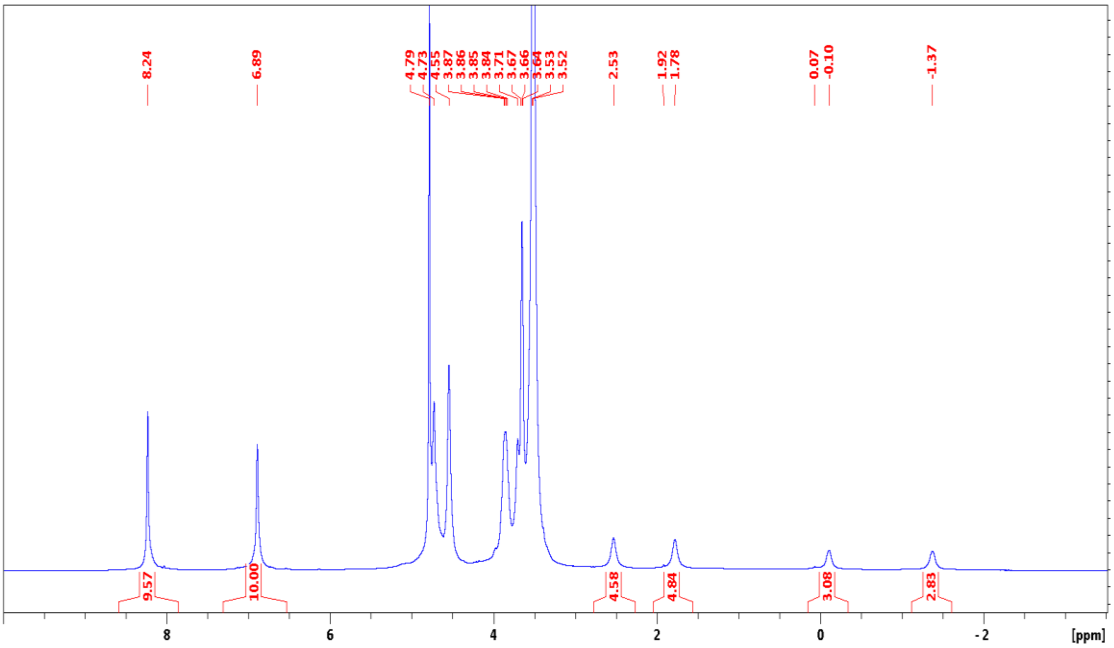

Figure S13. ^{1}H-NMR spectra (400 MHz, D_2O, 298K) of a 10:20 mM solution of **1** and **6a** respectively.

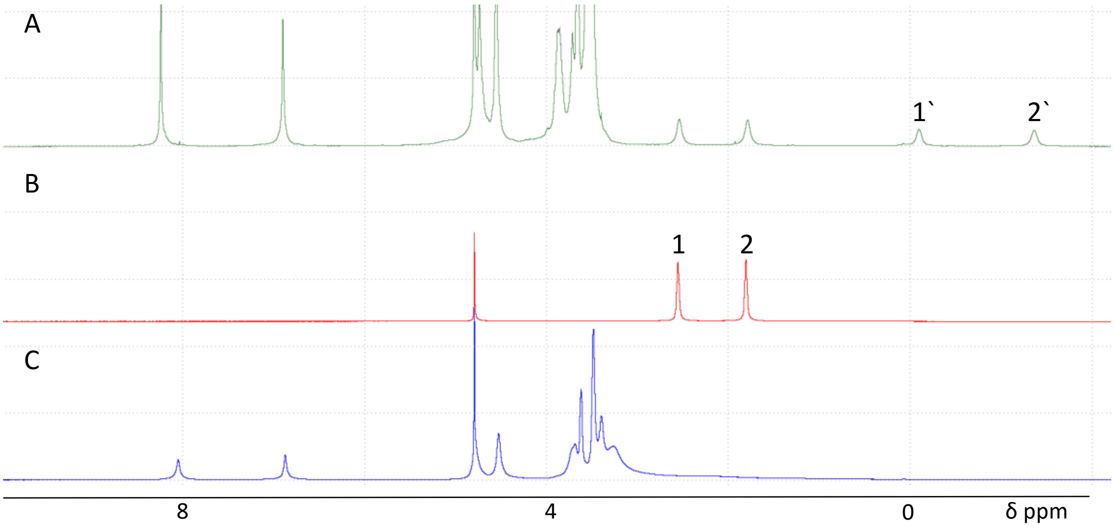

Figure S14. ^{1}H-NMR spectra (400 MHz, D_2O, 298K) of A) a 10:20 mM solution of **1** and **6a** respectively, B) **6a** (20 mM), and C) **1** (10 mM). Δδ: 1→1\`= 2.6 ppm, and 2→2\`= 3.2 ppm.

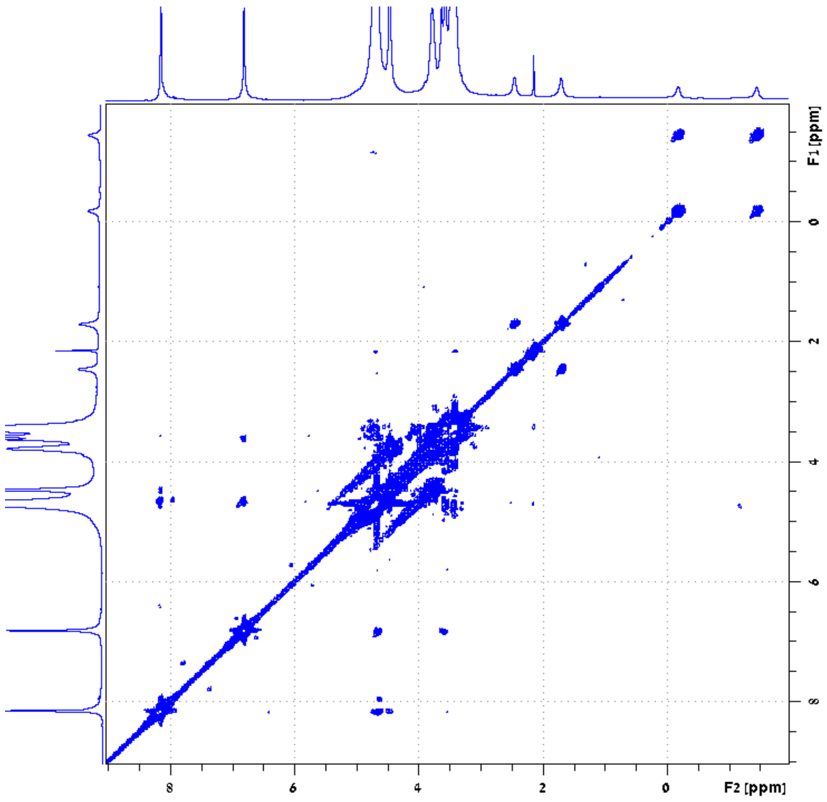

Figure S15. ^{1}H-COSY spectrum (400 MHz, D_2O, 298K) of a 10:20 mM solution of **1** and **6a**, respectively.

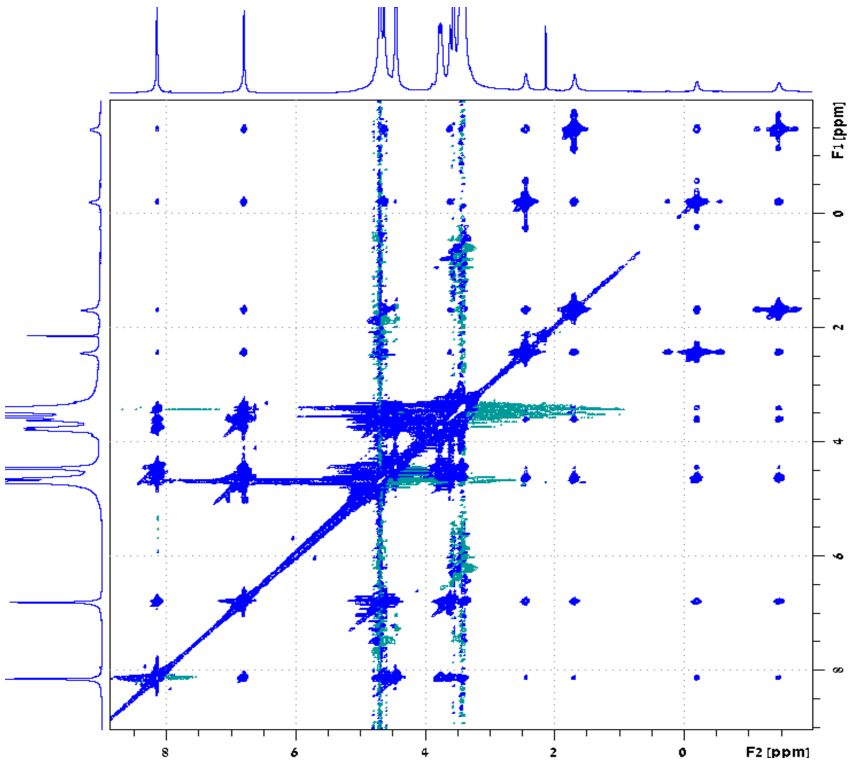

Figure S16. ^{1}H-NOESY spectrum (500 MHz, D_2O, 298K) of a 10:20 mM solution of **1** and **6a**, respectively.

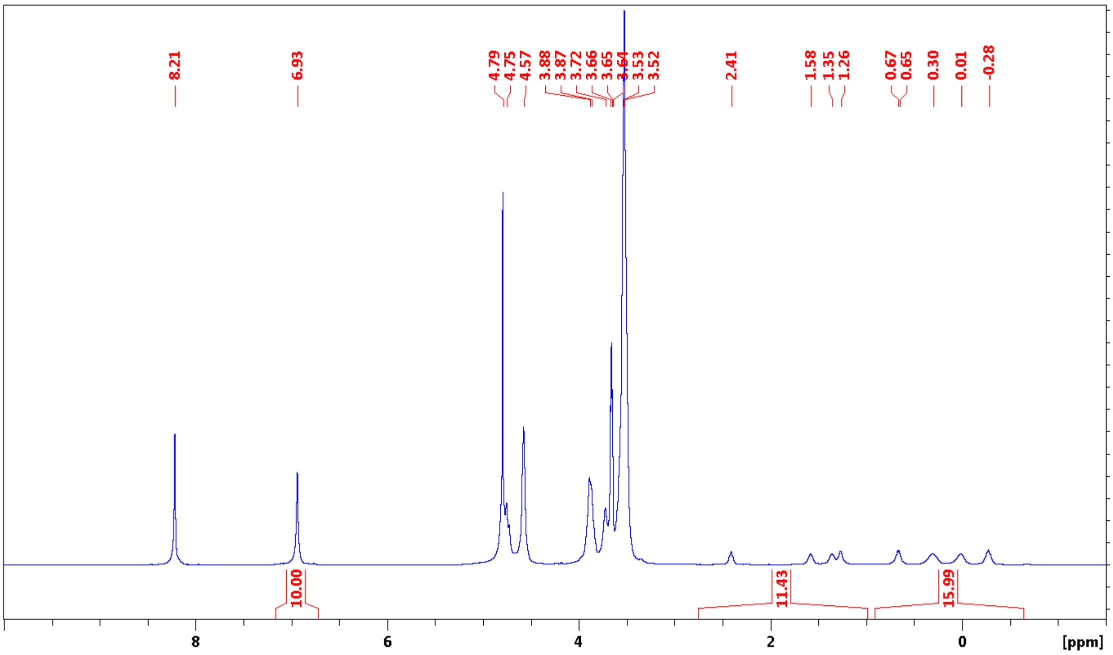

Figure S17. ^{1}H-NMR spectra (400 MHz, D_2O, 298K) of a 10:20 mM solution of **1** and **6b**, respectively.

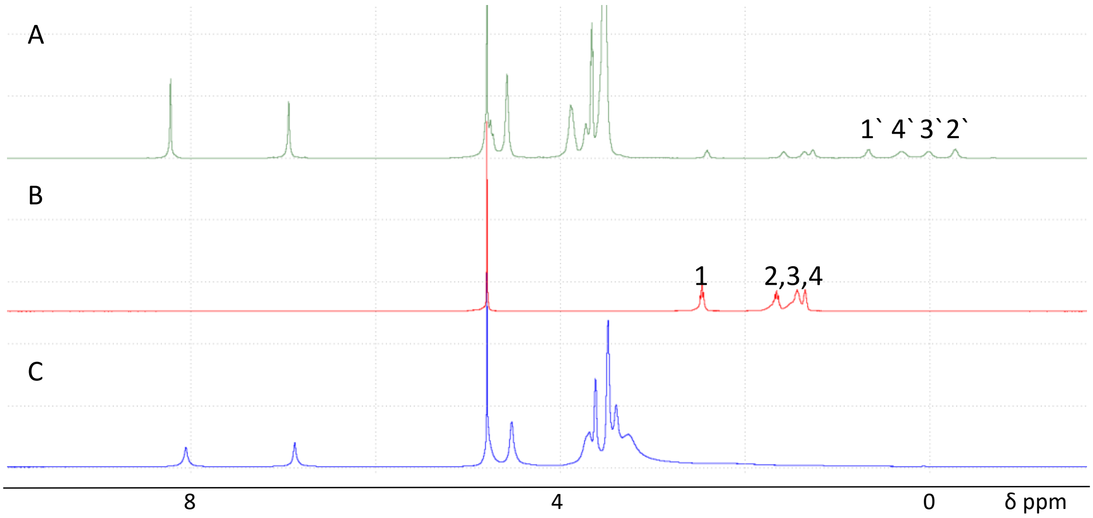

Figure S18. ^{1}H-NMR spectra in (400 MHz, D_2O, 298K) of A) a 10:20 mM solution of **1** and **6b**, respectively, B) **6b** (20 mM), and C) **1** (10 mM). Δδ: 1→1\`= 1.8 ppm, 2→2\`= 1.9 ppm, 3→3\`= 1.4 ppm, and 4→4\`= 1.0 ppm.

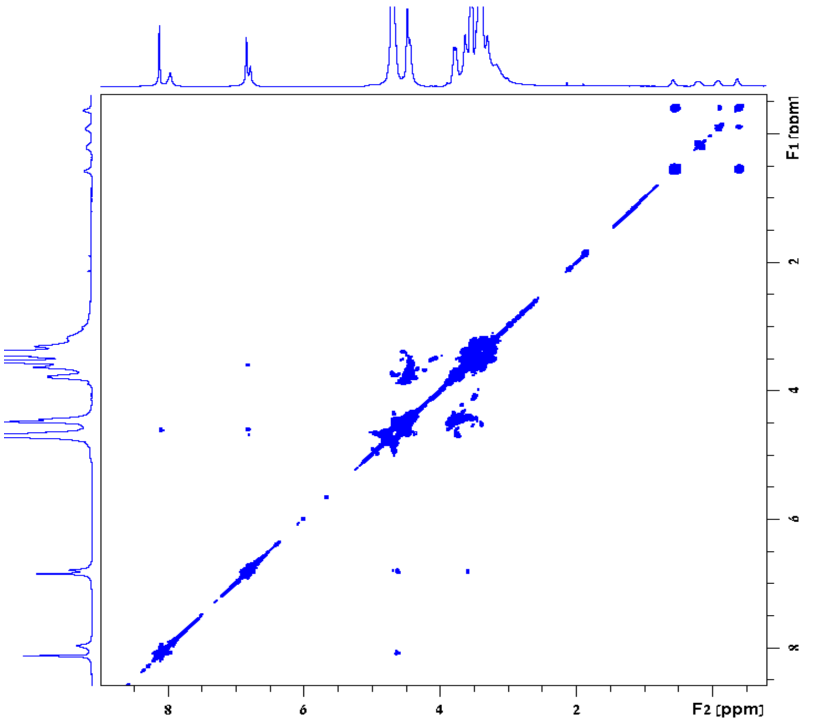

Figure S19. ^{1}H-COSY spectrum (500 MHz, D_2O, 298K) of a 10:10 mM solution of **1** and **6b**, respectively.

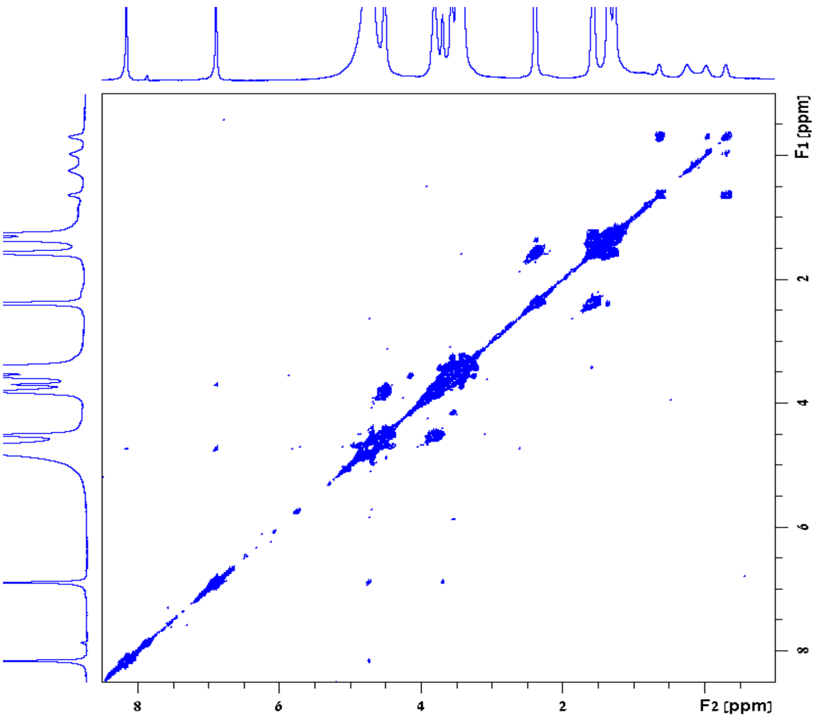

Figure S20. ^{1}H-COSY spectrum (400 MHz, D_2O, 298K) of a 10:20 mM solution of **1** and **6b**, respectively.

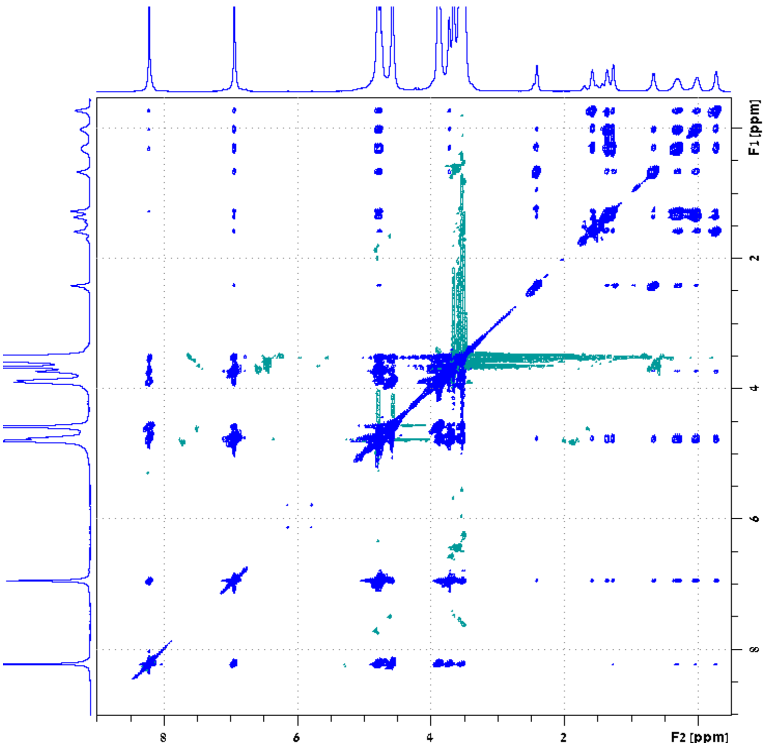

Figure S21. ^{1}H-NOESY spectrum (500 MHz, D_2O, 298K) of a 10:20 mM solution of **1** and **6b**, respectively.

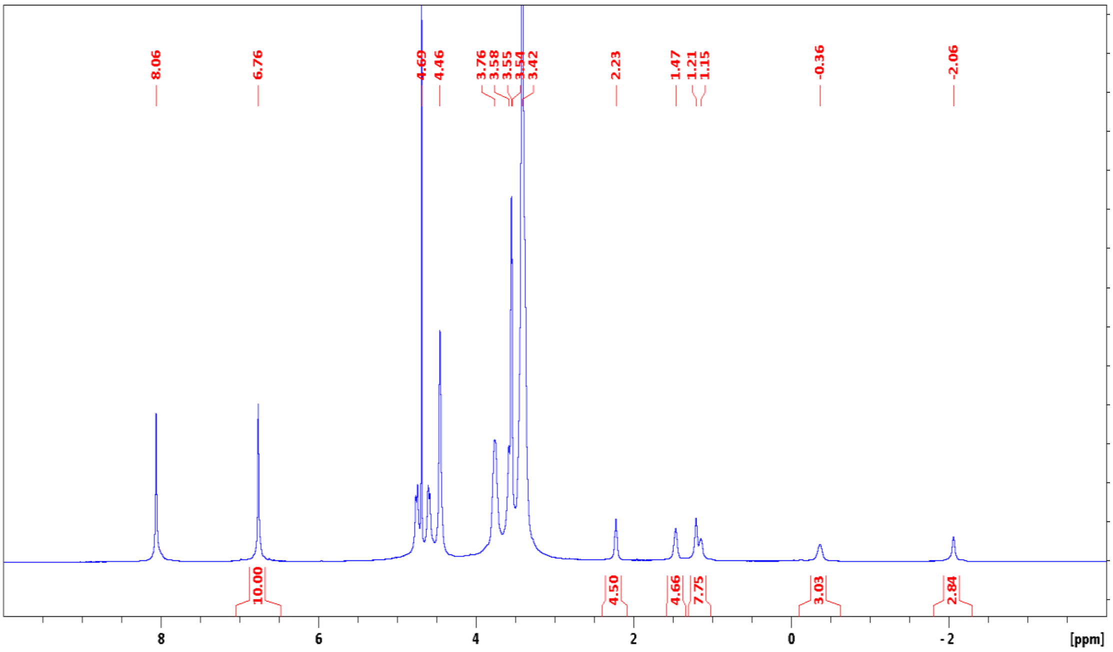

Figure S22. ^{1}H-NMR spectra (500 MHz, D_2O, 298K) of a 10:20 mM solution of **1** and **7a**, respectively.

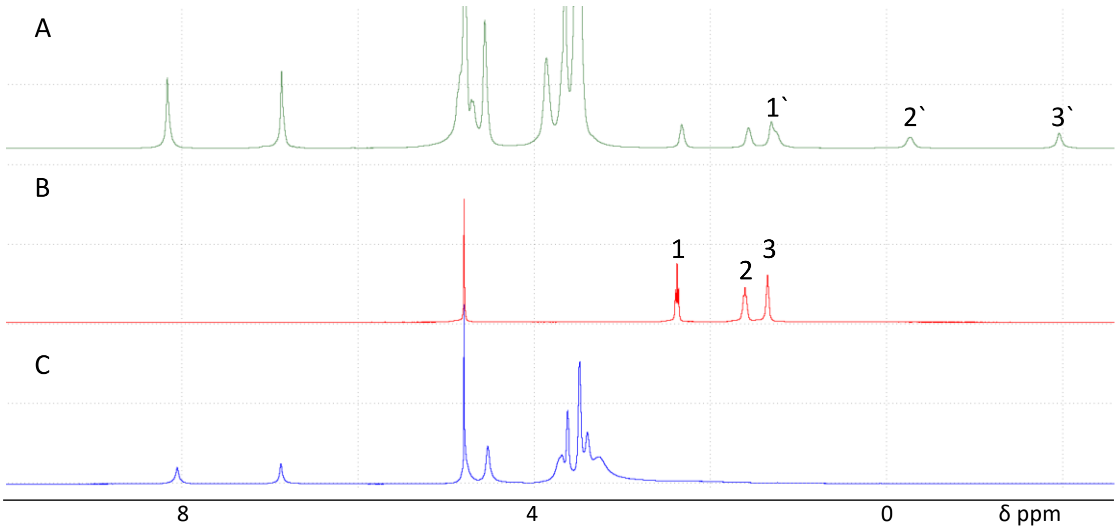

Figure S23. ^{1}H-NMR spectra in D_2O 400 MHz, 298K) of A) a 10:20 mM solution of **1** and **7a**, respectively, B) **7a** (20 mM). The sample was heated to 80 ^{0}C for 2h, and C) **1** (10 mM). Δδ: 1→1`= 1.0 ppm, 2→2`= 1.8 ppm, and 3→3`= 3.3 ppm.

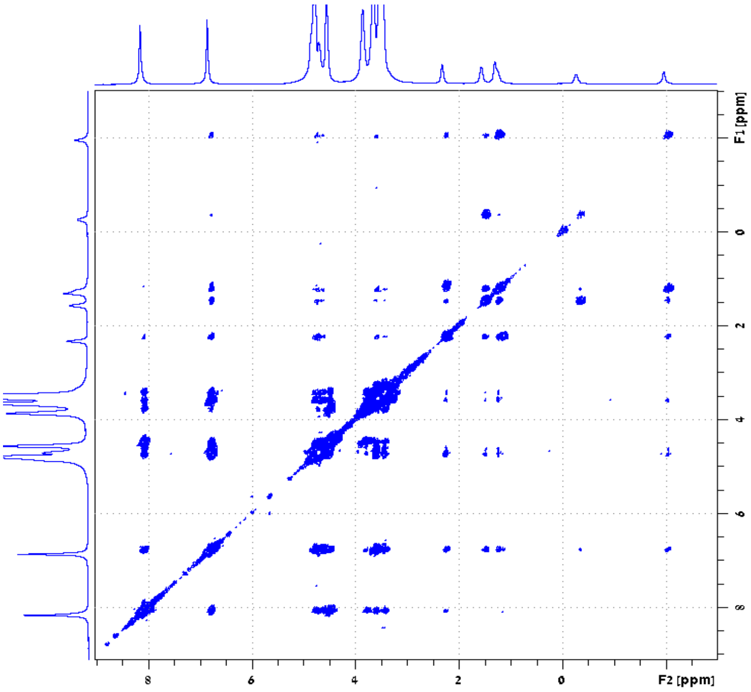

Figure S24. ^{1}H-NOESY spectrum (400 MHz, D_2O, 298K) of a 10:20 mM solution of **1** and **7a**, respectively.

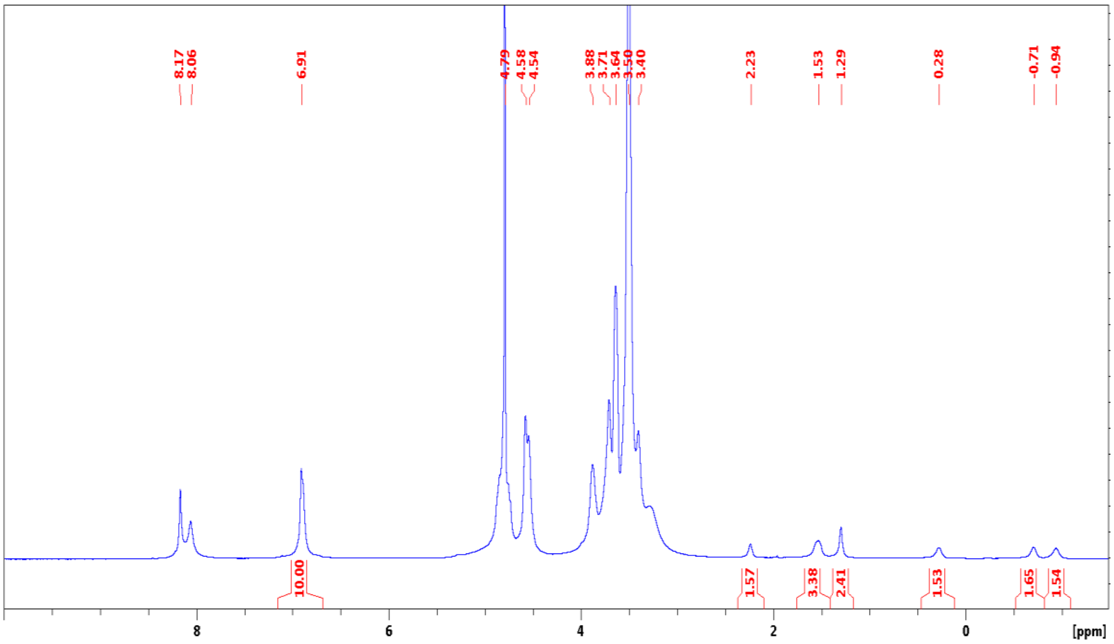

Figure S25. ^{1}H-NMR spectra (500 MHz, D_2O, 298K) a 10:20 mM solution of **1** and **7b**, respectively.

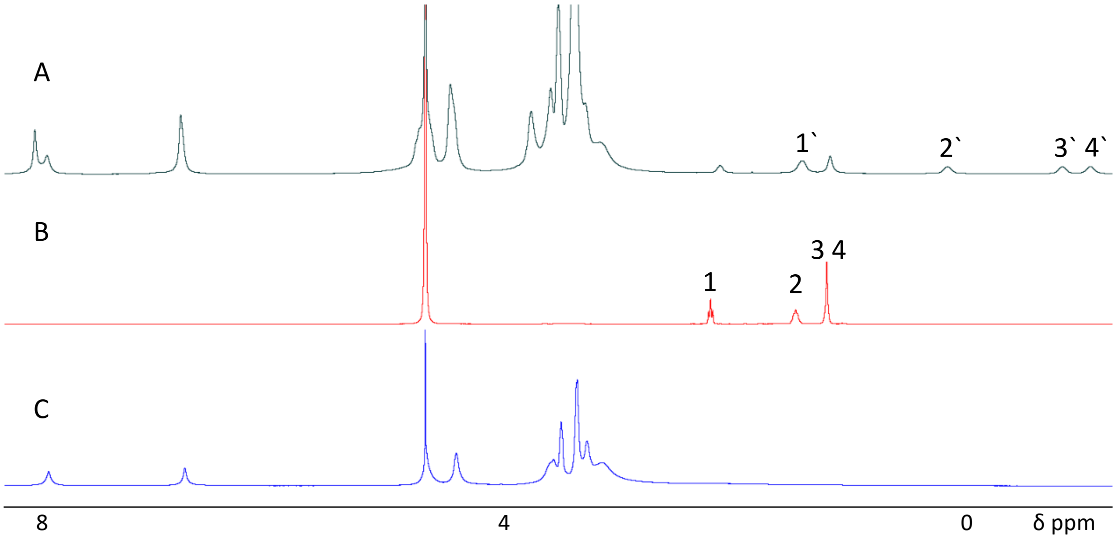

Figure S26. ^{1}H-NMR spectra in D_2O (400 MHz, 298K) of A) a 10:20 mM solution of **1** and **7b**, respectively, B) **7b**. and C) **1** (10 mM). Δδ: 1→1`= 0.8 ppm, 2→2`= 1.3 ppm, 3→3`= 2 ppm, and 4→4`= 2.3 ppm.

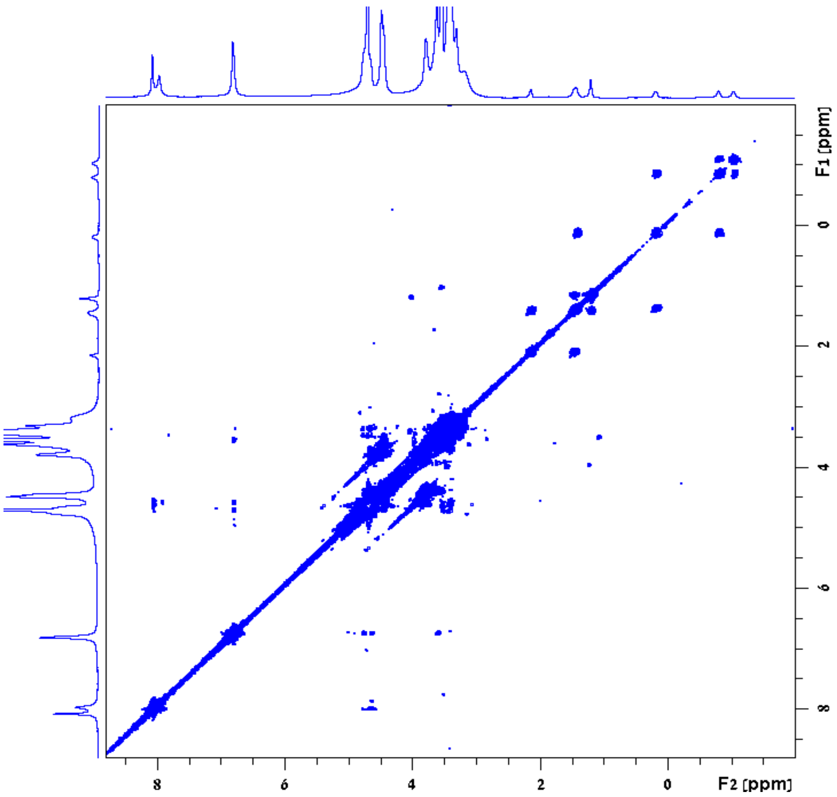

Figure S27. ^{1}H-COSY spectrum (500 MHz, D_2O, 298K) of a 10:20 mM solution of **1** and **7b**, respectively.

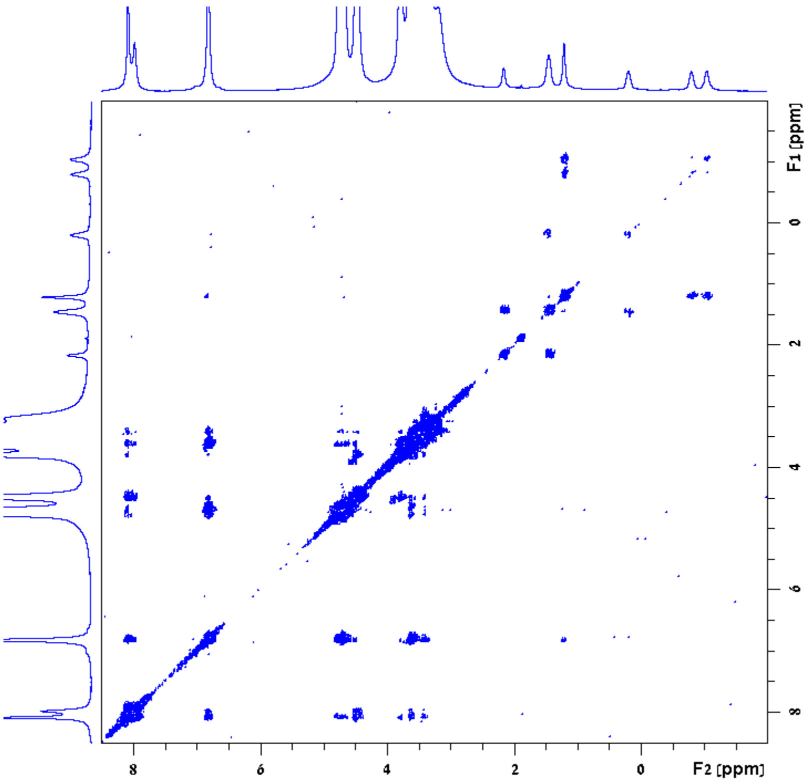

Figure S28. ^{1}H- NOESY spectrum (500 MHz, D_2O, 298K) of a 10:20 mM solution of **1** and **7b**, respectively.

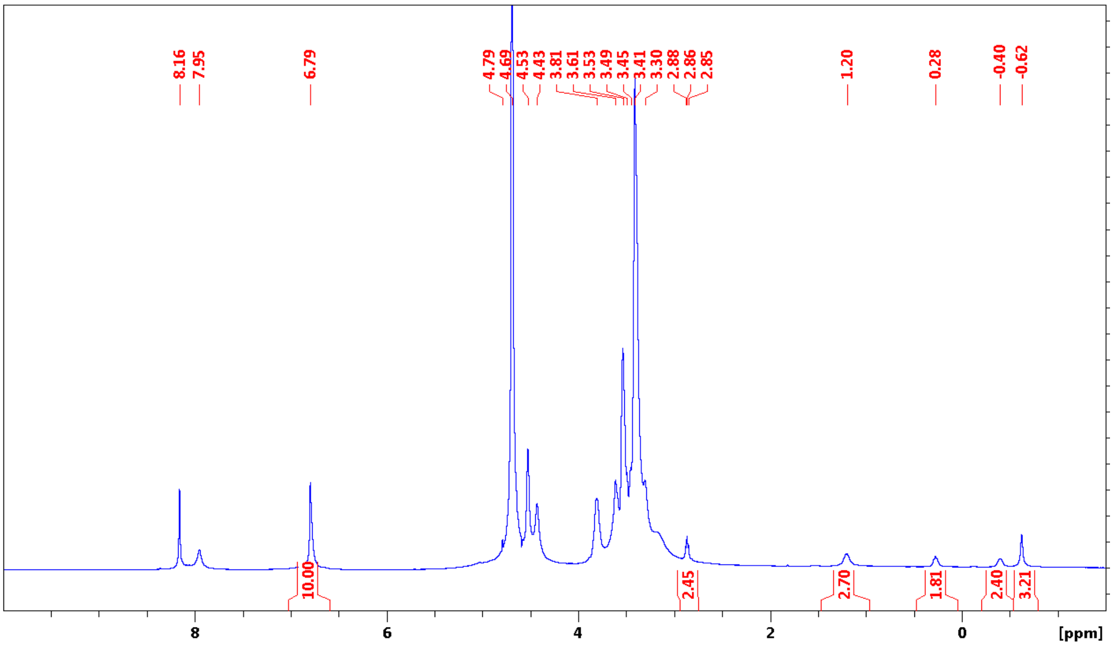

Figure S29. ^{1}H-NMR spectra (400 MHz, D_2O, 298K) of a 10:20 mM solution of **1** and **8a**, respectively. The sample was heated to 80 ^{0}C for 2h in.

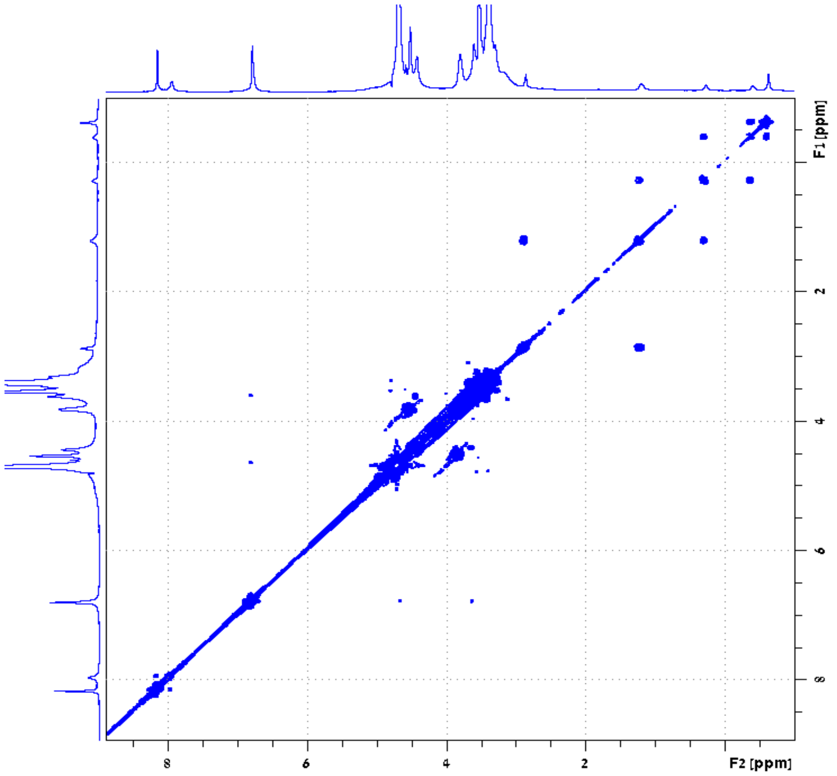

Figure S30. ^{1}H-COSY spectrum (500 MHz, D_2O, 298K) of a 10:20 mM solution of **1** and **8a**, respectively. The sample was heated to 80 ^{0}C for 2h.

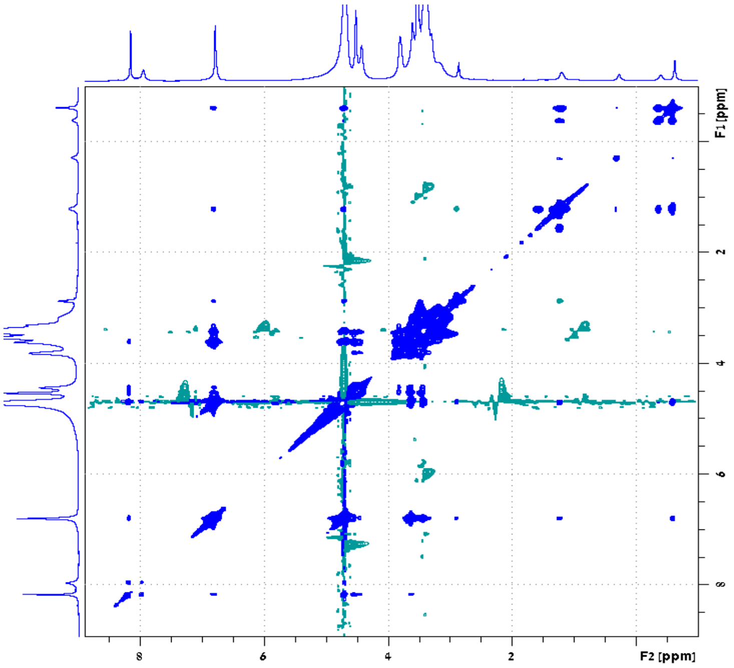

Figure S31. ^{1}H- NOESY spectrum (500 MHz, D_2O, 298K) of a 10:20 mM solution of **1** and **8a**, respectively. The sample was heated to 80 ^{0}C for 2h.

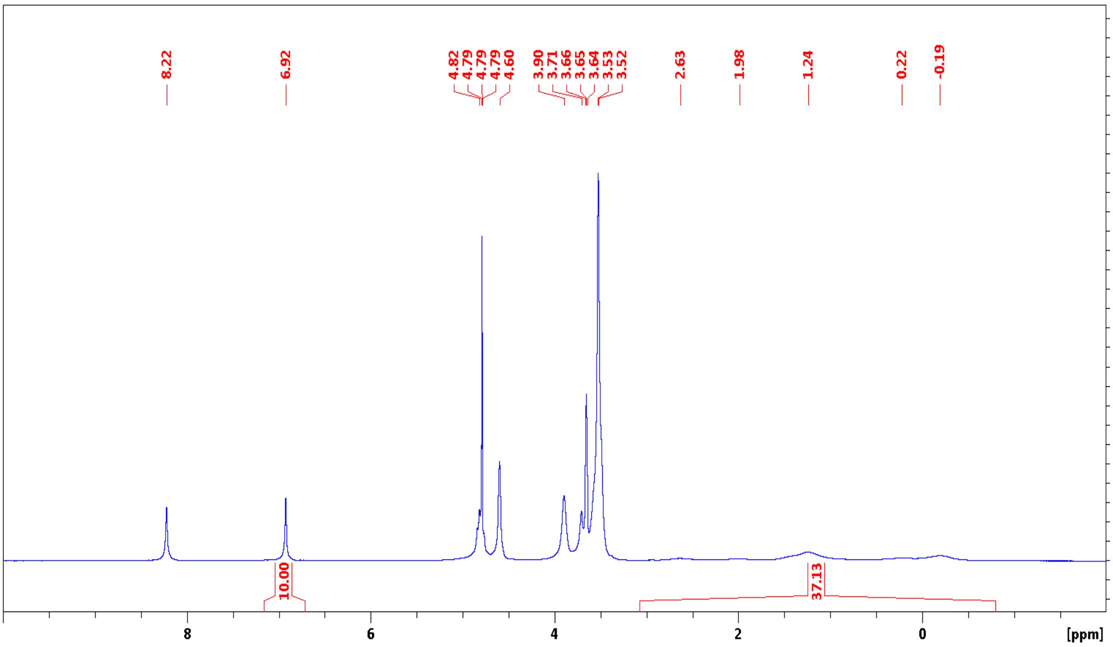

Figure S32. ^{1}H-NMR spectra (500 MHz, D_2O, 298K) of a 10:20 mM solution of **1** and **8b**, respectively. The sample was heated to 80 ^{0}C for 17h.

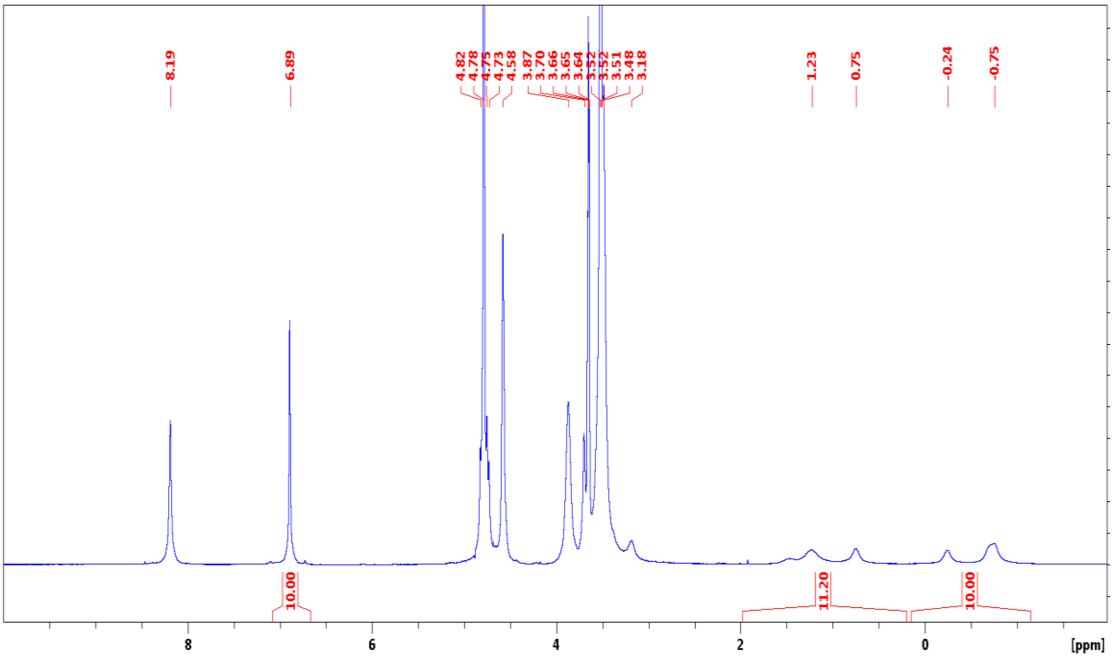

Figure S33. ^{1}H-NMR spectra (500 MHz, D_2O, 298K) of a 10:20 mM solution of **1** and **9**, respectively. The sample was heated to 85 ^{0}C for 24h.

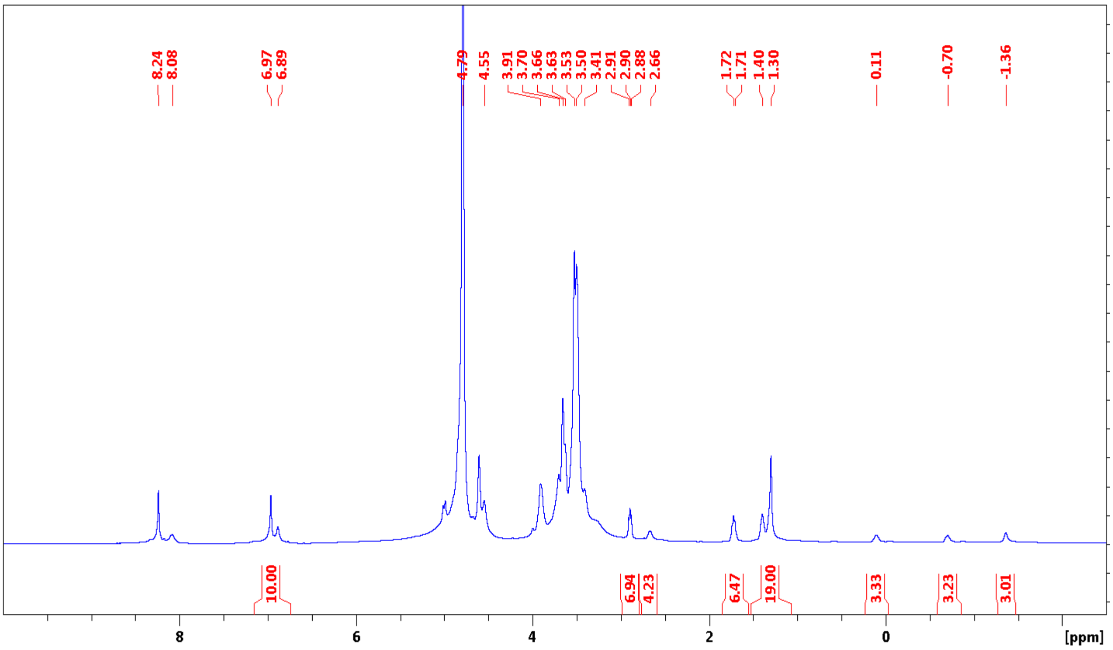

Figure S34. ^{1}H-NMR spectra (500 MHz, D_2O, 298K) of a 10:20 mM solution of **1** and **10**, respectively. The sample was heated to 85 ^{0}C for 24h.

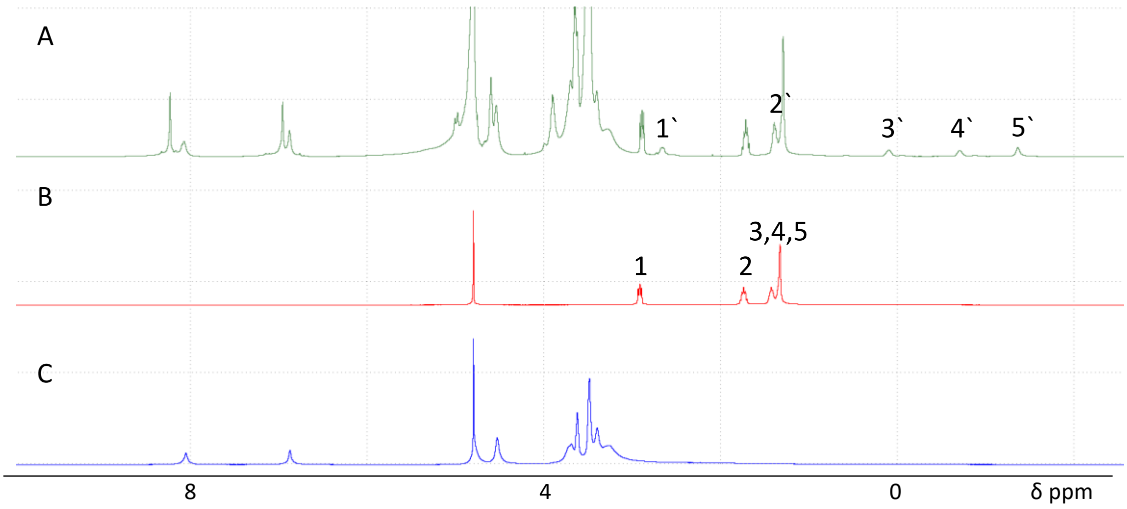

Figure S35. ^{1}H-NMR spectra (400 MHz, D_2O, 298K) of A) a 10:20 mM solution of **1** and **10**, respectively. The sample was heated to 85 ^{0}C for 24h, B) **10** after 2h at 80 ^{0}C (20 mM), and C) **1** (10 mM). $\Delta\delta_H$: 1→1`= 0.2 ppm, 2→2`= 0.4 ppm, 3→3`= 1.3 ppm, 4→4`=2.0 ppm, and 5→5`= 2.7 ppm.

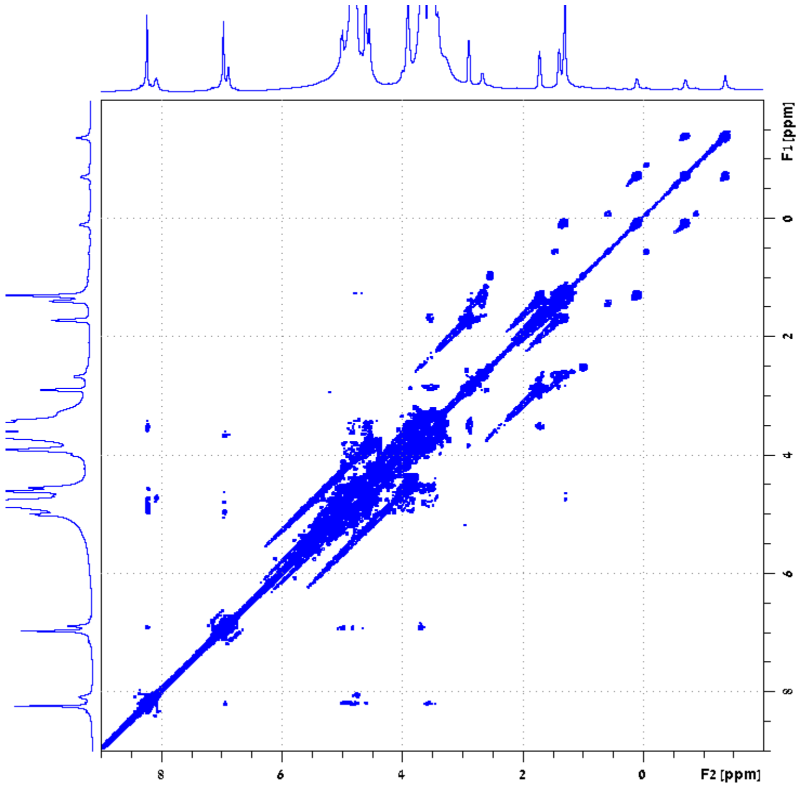

Figure S36. ^{1}H-COSY spectrum (500 MHz, D_2O, 298K) of a 10:20 mM solution of **1** and **10**, respectively. The sample was heated to 85 ^{0}C for 24h.

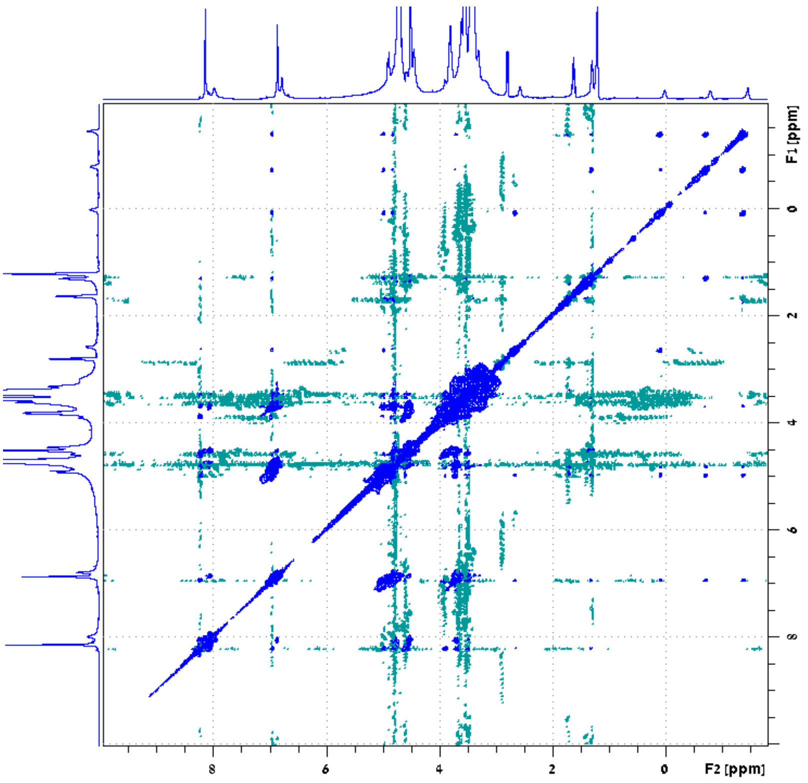

Figure S37. ^{1}H-NOESY spectrum (500 MHz, D_2O, 298K) of a 10:20 mM solution of **1** and **10**, respectively. The sample was heated to 85 ^{0}C for 24h.

6. ^{1}H-NMR of 1 and 12

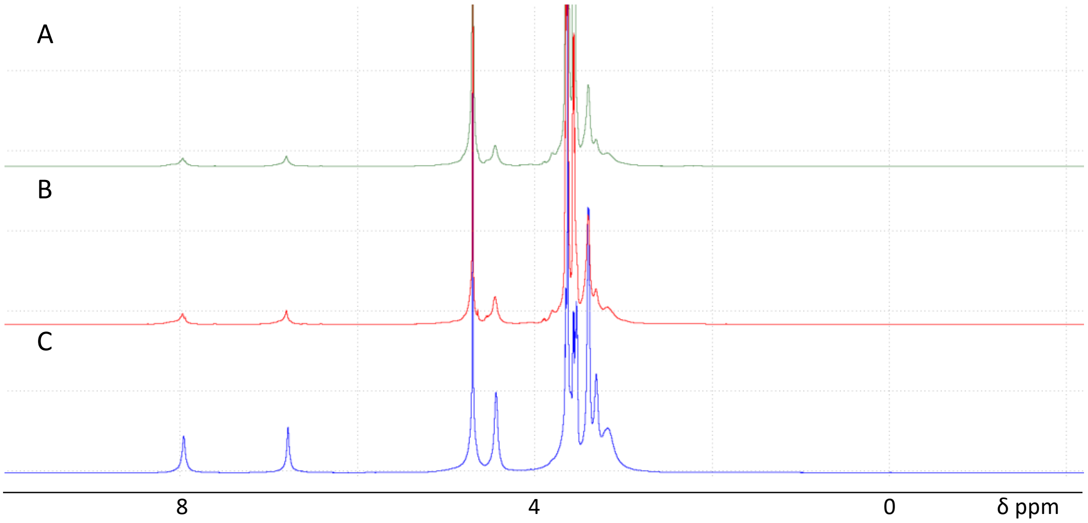

Figure S38. ^{1}H-NMR spectra (400 MHz, D_2O, 298K) of **1** (10 mM) and tetraethylene glycol at varying concentrations A) 20 mM, B) 60 mM C) 110 mM.

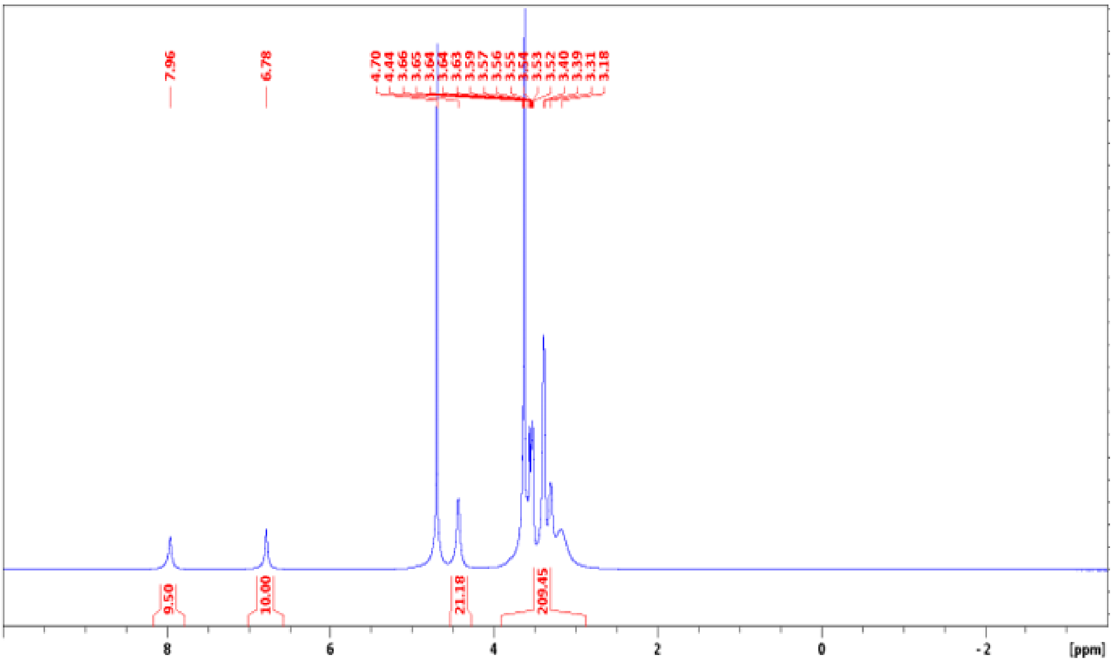

Figure S39. ^{1}H-NMR spectra (400 MHz, $CDCl_3$, 298K) of tetraethylene glycol (20 mM) and **1** (10 mM).

7. Diffusion NMR and DLS results

Table S1. Diffusion coefficient (D) at 298K for **1** in different solvents and at different concentrations.

**Sample Content**	**D value** $\times 10^{-5}\ cm^2s^{-1}$	
	**Solvent**	**Compound 1**
**1**[a] (0.4 mM)	1.91 ± 0.01	0.16 ± 0.01
**1**[a] (3 mM)	1.83 ± 0.01<br>1.91 ± 0.01[d]	0.14 ± 0.01<br>0.15 ± 0.01[d]
**1**[a] (50 mM)	1.36 ± 0.02<br>1.91 ± 0.01[d]	0.08 ± 0.01<br>0.11 ± 0.01[d]
**1**[b] (3 mM)	4.30 ± 0.07	0.57 ± 0.01
**1**[c] (3 mM)	2.42 ± 0.01	0.33 ± 0.01

[a] D_2O. [b] Acetone-d_6. [c] $CDCl_3$. [d] Corrected for change in water viscosity.

Table S2. Diffusion coefficient (D) (298K) in D_2O and a 1:1 solution of **1**:**12**.

**Sample Content**	**D value** $\times 10^{-5}\ cm^2s^{-1}$		
	**4.7 ppm**	**6.7 ppm**	**3.6 ppm**
**1:12**[a] **(5:5mM)**	1.73 ± 0.01<br>1.94 ± 0.01[b]	0.13 ± 0.01<br>0.15 ± 0.01[b]	0.41 ± 0.01<br>0.46 ± 0.01[b]
**12**[a] **(5mM)**	1.94 ± 0.01	N/A	0.54 ± 0.01

[a] D_2O; [b] after correction for change in viscosity

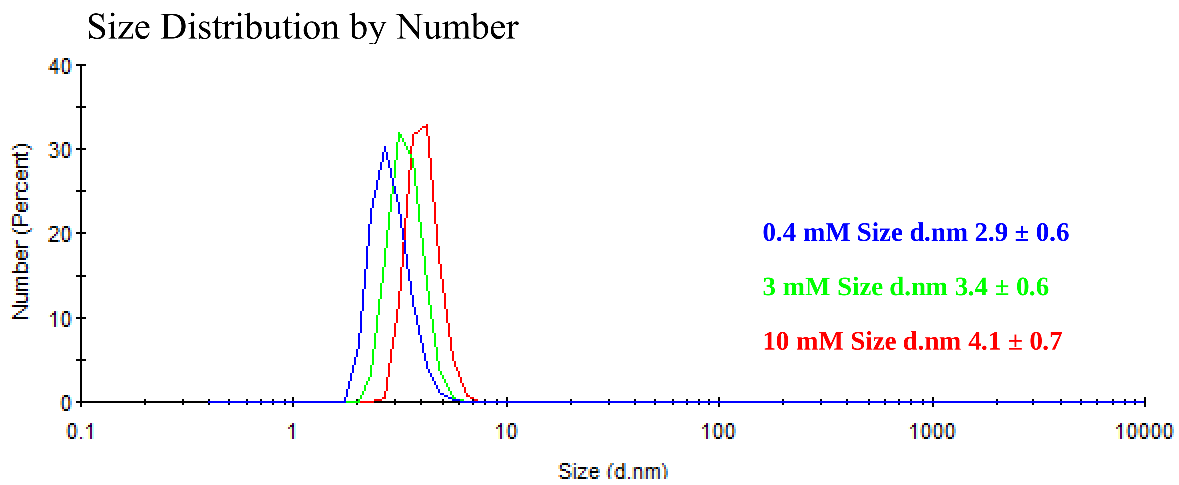

Figure S40. Size distribution by number of compound **1** in D_2O at different concentrations. The extracted radii were 1.4, 1.7 and 2.0 nm for the 0.4, 0.3 and 10.0 mM D_2O solution of **1**.

8. Size extraction from diffusion NMR

The hydrodynamic radii were calculated using the Stokes-Einstein equation (1) suitable for hard spheres

$$(1)\ r_s = \frac{K_B T}{6\pi\eta D}$$

Where K_B is the Boltzmann coefficient, T is the temperature, η is the viscosity of the solution and D is the diffusion coefficient as extracted form diffusion NMR experiments.

$$r_s(\mathbf{1}, 3\ \text{mM in}\ D_2O) = \frac{1.38 \times 10^{-23} m^2 kg s^{-1} K^{-1} \times 298K}{6\pi\ \times 8.90 \times 10^{-4} Pa.s \times 0.15 \times 10^{-9} m^2 s^{-1}}$$

$$r_s(\mathbf{1}, 3\ \text{mM}\ in\ D_2O) = 1.63\ nm$$

$$r_s(\mathbf{1}, 3\ \text{mM}\ \ in\ CDCl_3) = \frac{1.38 \times 10^{-23} m^2 kg s^{-1} K^{-1} \times 298K}{6\pi\ \times 5.42 \times 10^{-4} Pa.s \times 0.33 \times 10^{-9} m^2 s^{-1}}$$

$$r_s(\mathbf{1}, 3\ \text{mM in}\ CDCl_3) = \ 1.22\ nm$$

$$r_s(\mathbf{1}, 3\ \text{mM in Acetone}) = \frac{1.38 \times 10^{-23} m^2 kg s^{-1} K^{-1} \times 298K}{6\pi\ \times 3.16 \times 10^{-4} Pa.s \times 0.57 \times 10^{-9} m^2 s^{-1}}$$

$$r_s(\mathbf{1}, 3\ \text{mM in Acetone}) = \ 1.21\ nm$$

$$r_s(\mathbf{1}, 0.4\ \text{mM}\ in\ D_2O) = \frac{1.38 \times 10^{-23} m^2 kg s^{-1} K^{-1} \times 298K}{6\pi\ \times 8.90 \times 10^{-4} Pa.s \times 0.16 \times 10^{-9} m^2 s^{-1}}$$

$$r_s(\mathbf{1}, 0.4\ \text{mM}\ in\ D_2O) = \ 1.53\ nm$$

9. Estimation of the association constants (K_as) of 1 with 6a and 6b

This data is based on simple integration of a fully relaxed ^{1}H-NMR spectra of the mixture assuming the formation of 1:1 host-guest complex. The error is estimated to be ~30%.

$$k_a(\ 1{:}1\ \text{mM}\ \mathbf{1}{:}\ \mathbf{6a}\ ) = \frac{0.57\ mM \times 10^{-3}}{0.43\ mM \times 10^{-3} \times\ 0.43\ mM \times 10^{-3}} = 3.1 \times 10^3 M^{-1}$$

$$k_a(\ 1{:}1\ \text{mM}\ \mathbf{1}{:}\ \mathbf{6b}\ ) = \frac{0.8\ mM \times 10^{-3}}{0.2\ mM \times 10^{-3} \times 0.2\ mM \times 10^{-3}} = 2.0 \times 10^4\ M^{-1}$$

10. Extraction experiments

For the extraction experiments the following 10mM stock solutions were prepared: 1) 3mL of compound **1** in D_2O; 2) One mL of a 1:1 solution of compounds **6a** and **6b** in C_6D_6; 3) a 1:1 solution of compounds **6a** and **11** C_6D_6, and 4) a 1:1 solution of compounds **6b** and **11** C_6D_6.

Then 1mL of solution (1) was mixed with 1mL of solutions (2), (3) or (4) and vigorously stirred for 90 minutes. The magnet stirring was stopped and the D_2O phase of each mixture was separated and measured by ^{1}H-NMR at 500MHz. For the attempt of recycling host **1,** the 1mL D_2O phase of the previous experiment was mixed 1mL solution of C_6D_6 and the mixture was vigorously stirred for 90 minutes and then separated. This procedure was repeated twice for each solution and then the D_2O phase of each solution was re-examined by ^{1}H-NMR at 500MHz. The results are summarized in Table S3.

A second series of extraction experiments were performed in the same way, but there a 5mM D_2O solutions of **1** and 5mM C_6D_6 solutions of **6a**, **6b** and **11** were used. The results are summarized in Table S3.

Table S3. Results of the extraction experiments. Percentage was calculated from ^{1}H-NMR integration.

Experiment stage	Sample Content	Extracted compound		
		**6a**	**6b**	**11**
First extraction after 90 minutes.	**1**[a]:**6a**[a]:**6b**[a]	77 %	10 %	-
	**1**[a]:**6a**[a]:**11**[a]	67 %	-	10 %
	**1**[a]:**6b**[a]:**11**[a]	-	22 %	22 %
	**1**[b]:**6a**[b]	69 %	-	-
	**1**[b]:**6b**[b]	-	18 %	-
	**1**[b]:**11**[b]	-	-	14 %
First washing cycle	**1**[a]:**6a**[a]:**6b**[a]	63 %	4 %	-
	**1**[a]:**6a**[a]:**11**[a]	48 %	-	1 %
	**1**[a]:**6b**[a]:**11**[a]	-	8 %	7 %
Second washing cycle	**1**[a]:**6a**[a]: **6b**[a]	42 %	-	-
	**1**[a]:**6a**[a]:**11**[a]	41 %	-	-
	**1**[a]:**6b**[a]:**11**[a]	-	-	-

[a] 10 mM. [b] 5 mM.

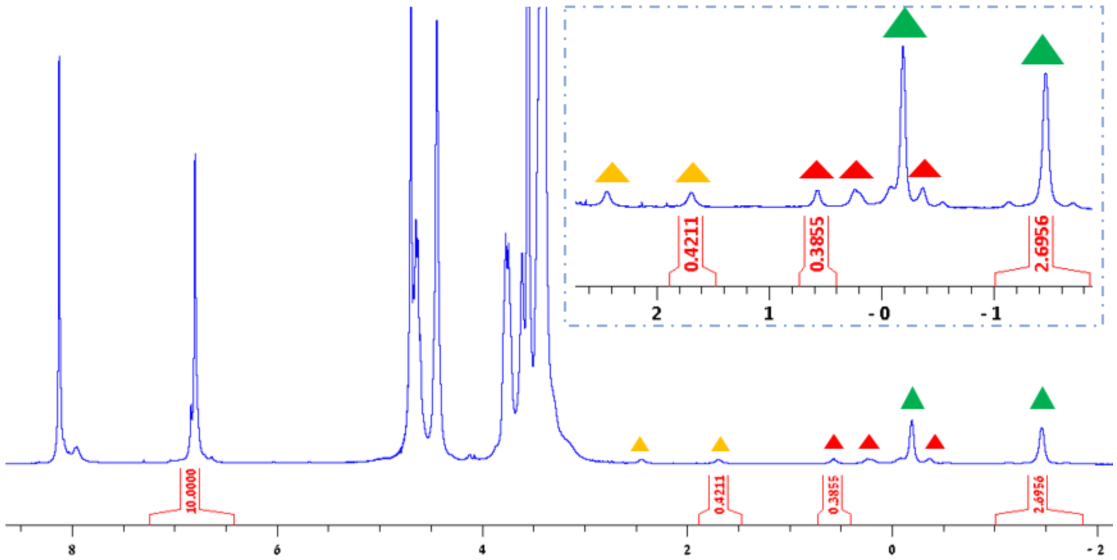

Figure S41. ^{1}H-NMR spectra (D_2O, 500 MHz, 298K) after first extraction of a 10mM solution of **6a** and **6b,** by 10mM D_2O solution of **1**. Yellow signals represent free **6a**, green signals represent encapsulated **6a**, red signals represent encapsulated **6b**.

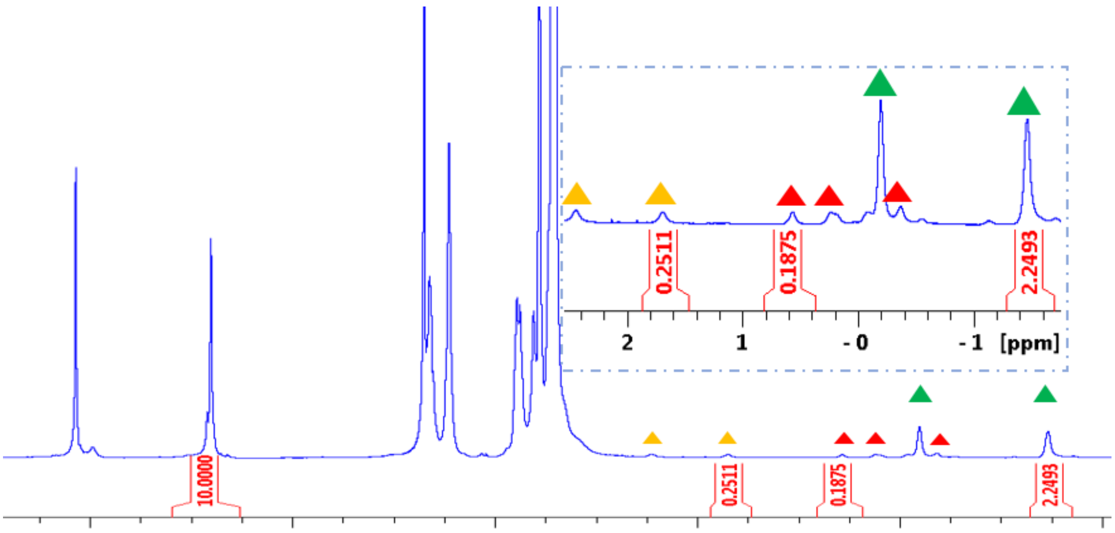

Figure S42. ^{1}H-NMR spectra (D_2O, 500 MHz, 298K) following the first washing cycle with fresh C_6D_6 of the solution shown in Figure S41. Yellow signals represent free **6a**, green signals represent encapsulated **6a**, red signals represent encapsulated **6b**.

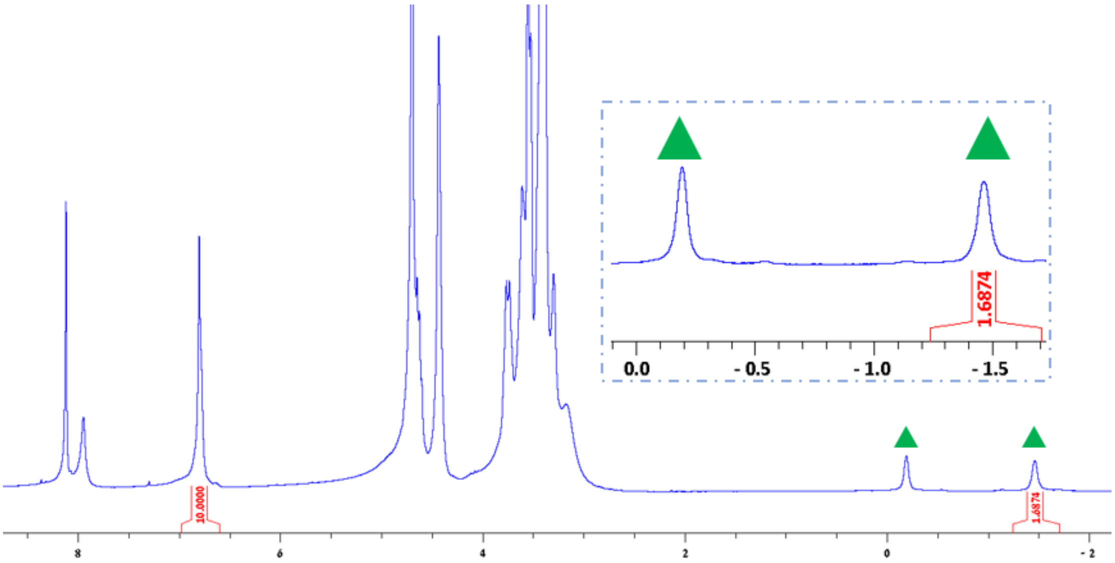

Figure S43. ^{1}H-NMR spectra (D_2O, 500 MHz, 298K) following the second washing cycle with fresh C_6D_6 of the solution shown in Figure S42. Green signals represent encapsulated **6a**.

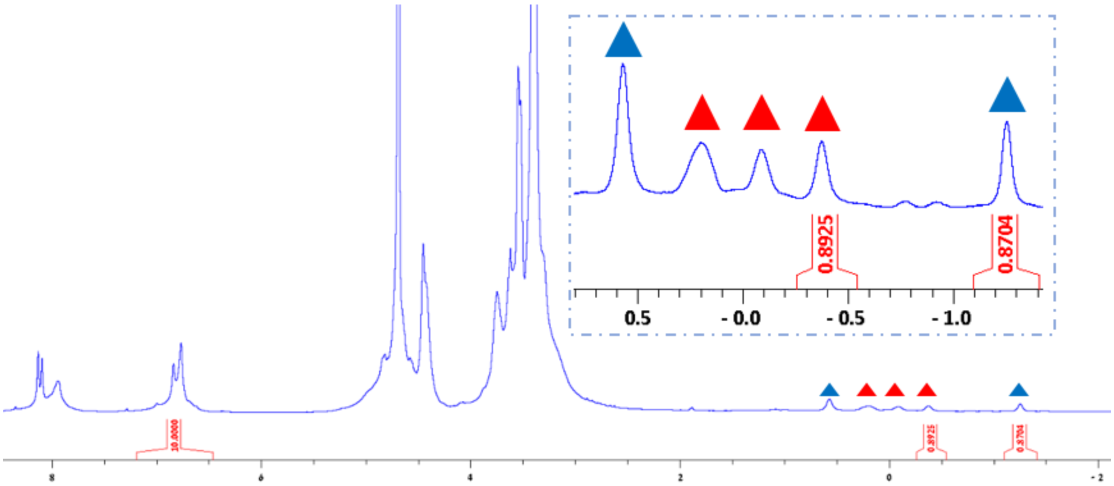

Figure S44. ^{1}H-NMR spectra (D_2O, 500 MHz, 298K) after first extraction of a of a 10mM solution of **6b** and **1,** by 10mM D_2O solution of **1**. Red signals represent encapsulated **6b**, blue signals represent encapsulated **11**.

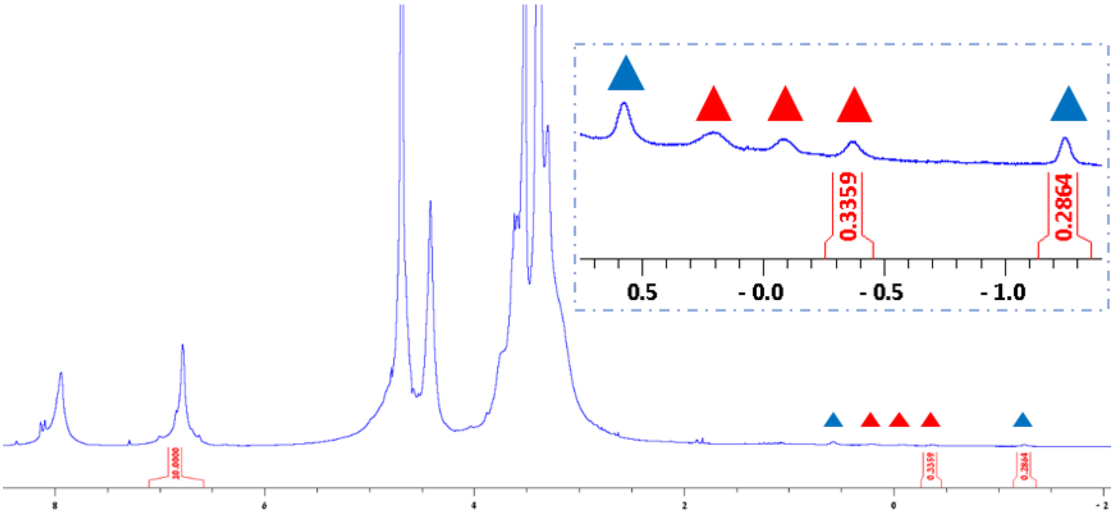

Figure S45. ^{1}H-NMR spectra (D_2O, 500 MHz, 298K) following the first washing cycle with fresh C_6D_6 of the solution shown in Figure S44. Red signals represent encapsulated **6b**, blue signals represent encapsulated **11**.

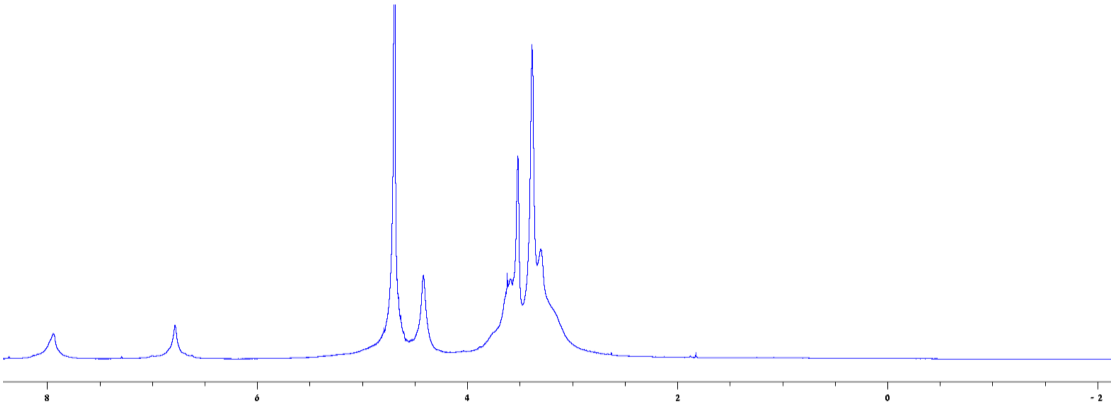

Figure S46. ^{1}H-NMR spectra (D_2O, 500 MHz, 298K) following the second washing cycle with fresh C_6D_6 of the solution shown in Figure S45.

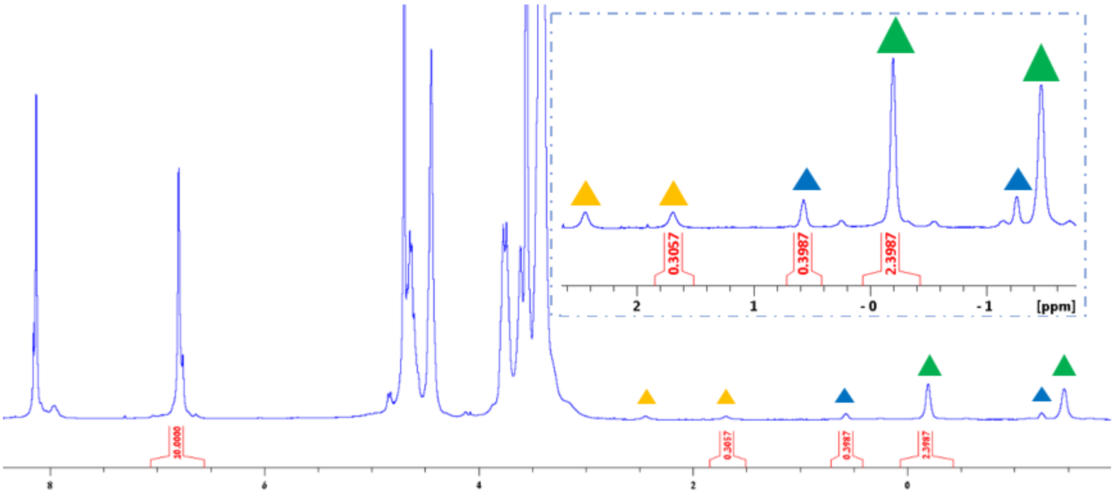

Figure S47. ^{1}H-NMR spectra (D_2O, 500 MHz, 298K) after first extraction of a 10mM solution of **6a** and **11**, by 10mM D_2O solution of **1**. Yellow signals represent free **6a**, green signals represent encapsulated **6a**, blue signals represent encapsulated **11**.

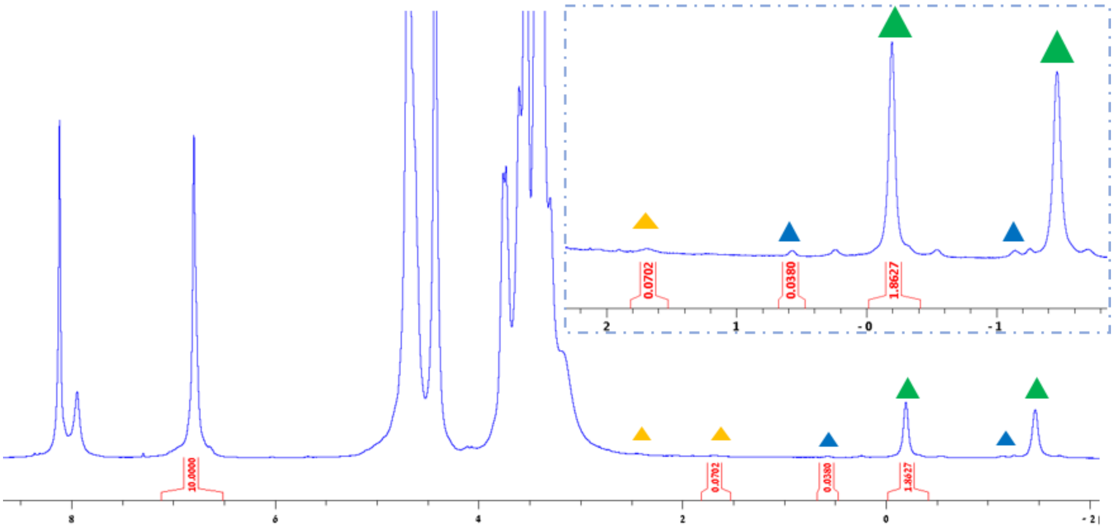

Figure S48. ^{1}H-NMR spectra (D_2O, 500 MHz, 298K) following the first extraction cycle with fresh C_6D_6 of the solution shown in Figure S47. Yellow signals represent free **6a**, green signals represent encapsulated **6a**, blue signals represent encapsulated **11**.

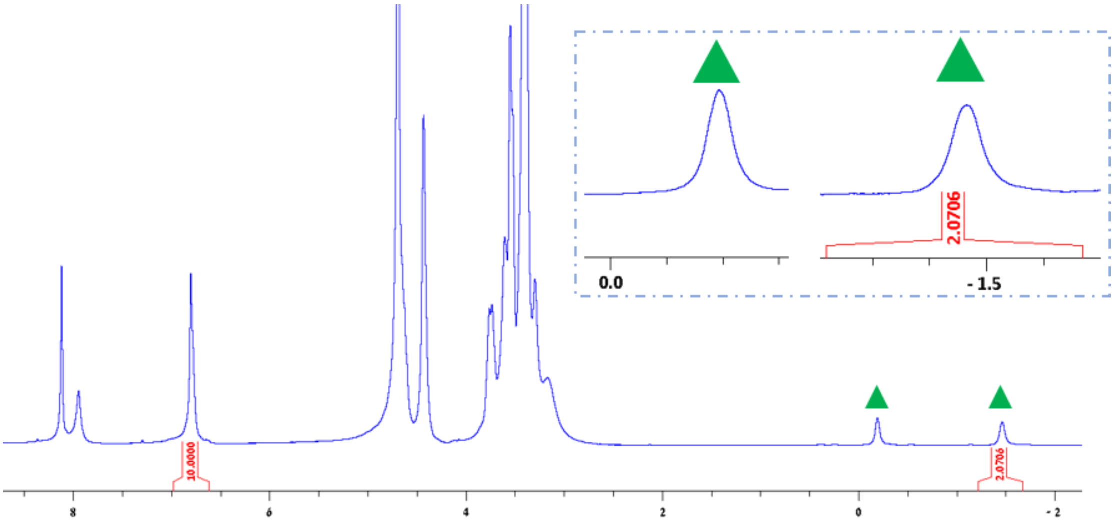

Figure S49. ^{1}H-NMR spectra (D_2O, 500 MHz, 298K) following the second extraction cycle with fresh C_6D_6 of the solution shown in Figure S48. Green signals represent encapsulated **6a**.

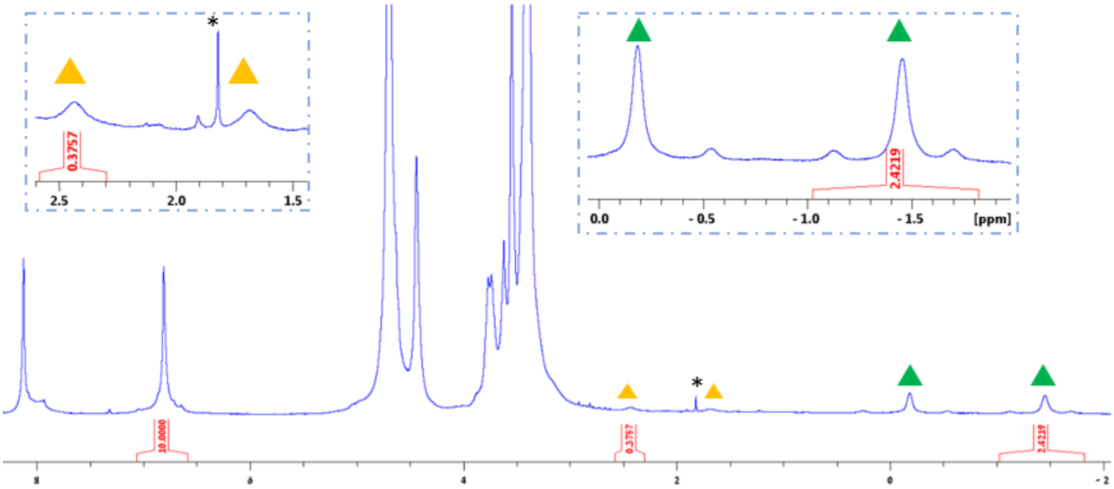

Figure S50. ^{1}H-NMR spectra (D_2O, 500 MHz, 298K) after first extraction of a 5mM C_6D_6 solution of **6a** by 5mM D_2O solution of **1**. Yellow signals represent free **6a**, green signals represent encapsulated **6a**. * Acetone residues.

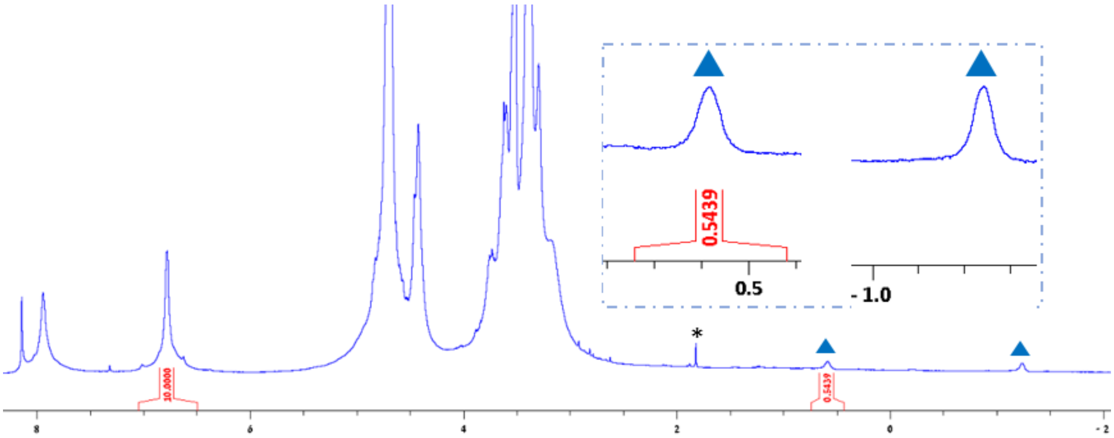

Figure S51. ^{1}H-NMR spectra (D_2O, 500 MHz, 298K) after first extraction of a 5mM C_6D_6 solution of **11** by 5mM D_2O solution of **1**. Blue signals represent encapsulated **11**.

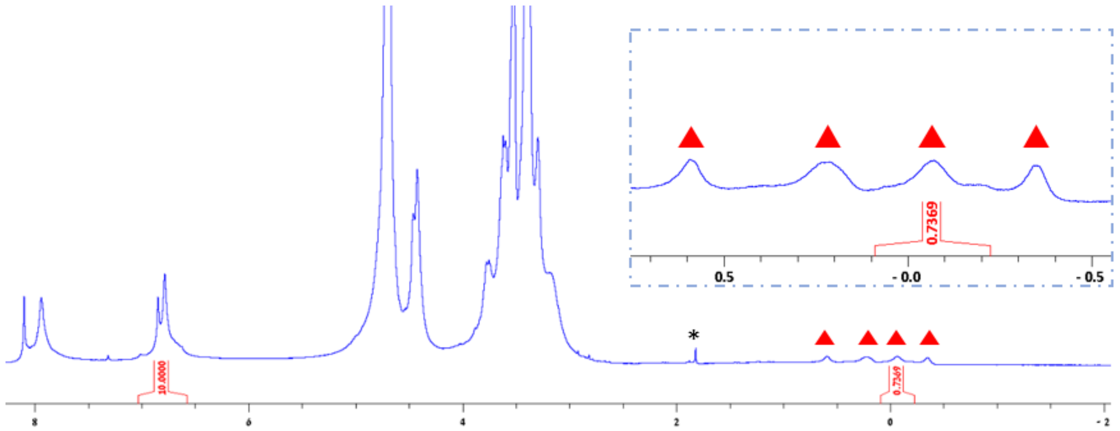

Figure S52. ^{1}H-NMR spectra (D_2O, 500 MHz, 298K after first extraction of a 5mM C_6D_6 solution of **6b** by 5mM D_2O solution of **1**. Red signals represent encapsulated **6b**. * Acetone residues

10. References

1. S. J. Gibbs, C. S. Johnson Jr., *J. Magn. Reson*., 1991, **93**, 395-402.
2. I. Nierengarten, M. Nothisen, D. Sigwalt, T. Biellmann, M. Holler, J.-S. Remy, J.-F. Nierengarten, *Chem. Eur. J.*, 2013, **19**, 17552.
3. Z-X. Jiang, Y. Feng and Y. Bruce Yu, *Chem. Commun*., 2011, **47**, 7233-7235.
